# Morphometric characteristics of tibial nerve and their relationship with age

**DOI:** 10.1093/braincomms/fcaf267

**Published:** 2025-07-07

**Authors:** Shahram Oveisgharan, Armand Collin, Jingyun Yang, Sue E Leurgans, Veronique VanderHorst, David A Bennett, Julien Cohen-Adad, Osvaldo Delbono, Aron S Buchman

**Affiliations:** Rush Alzheimer’s Disease Center, Rush University Medical Center, Chicago, IL, USA; Department of Neurological Sciences, Rush University Medical Center, Chicago, IL, USA; Institute of Biomedical Engineering, Polytechnique Montreal, Montreal, QC, Canada; Mila—Quebec AI Institute, Montreal, QC, Canada; Rush Alzheimer’s Disease Center, Rush University Medical Center, Chicago, IL, USA; Department of Neurological Sciences, Rush University Medical Center, Chicago, IL, USA; Rush Alzheimer’s Disease Center, Rush University Medical Center, Chicago, IL, USA; Department of Neurological Sciences, Rush University Medical Center, Chicago, IL, USA; Division of Movement Disorders, Department of Neurology, Beth Israel Deaconess Medical Center and Harvard Medical School, Boston, MA, USA; Rush Alzheimer’s Disease Center, Rush University Medical Center, Chicago, IL, USA; Department of Neurological Sciences, Rush University Medical Center, Chicago, IL, USA; Institute of Biomedical Engineering, Polytechnique Montreal, Montreal, QC, Canada; Mila—Quebec AI Institute, Montreal, QC, Canada; Functional Neuroimaging Unit, CRIUGM, University of Montreal, Montreal, QC, Canada; Centre de Recherche du CHU Sainte-Justine, Université de Montréal, Montreal, QC, Canada; Department of Internal Medicine, Gerontology and Geriatric Medicine, Wake Forest University School of Medicine, Winston-Salem, NC, USA; Rush Alzheimer’s Disease Center, Rush University Medical Center, Chicago, IL, USA; Department of Neurological Sciences, Rush University Medical Center, Chicago, IL, USA

**Keywords:** aging, tibial nerve, morphometry, myelinated nerve fibres

## Abstract

Peripheral nerve comprises a crucial component of the distributed motor/sensory system. However, there is a paucity of data on peripheral nerve morphology derived from large numbers of older adults. This study aimed to quantify the morphometric characteristics of myelinated nerve fibres of the tibial nerve obtained from deceased community-dwelling older adults and examine their association with age. The tibial nerves were obtained from consecutive autopsies of older adults without a history of diabetes who were participants of the Rush Memory and Aging Project, an ongoing longitudinal clinical-autopsy study. A nerve fascicle, obtained from a fixed popliteal segment of the tibial nerve, was separated from the blood vessels and adipose tissue for postmortem examination under an optical microscope. Morphometric characteristics of the myelinated nerve fibres were automatically segmented and quantified using our open-source software *AxonDeepSeg*. The participants (*N* = 140) had a mean age of 92.0 years (SD = 5.4) at death, and 72.1% (*N* = 101) were women. We examined 754 247 myelinated nerve fibres, with an average 5387 (SD = 3436) nerve fibres per participant. The average diameter of myelinated nerve fibres was 4.9 µm (SD = 3.1), axon diameter was 2.0 µm (SD = 1.4), myelin thickness was 1.4 µm (SD = 0.96) and the *g*-ratio (ratio of axon diameter to myelinated nerve fibre diameter) was 0.45 (SD = 0.17). The relationship between axon diameter and myelin thickness was nonlinear. Myelin was thicker in larger axons up to a diameter of 8 µm, beyond which myelin thickness plateaued. Older age at death was associated with smaller myelinated nerve fibres, smaller axons and thinner myelin. However, age at death was not correlated with myelinated nerve fibre density and was not associated with the average of *g*-ratio. The association between older age and smaller myelinated nerve fibres was largely attributable to a lower percentage of myelinated nerve fibres >8 µm. We conclude that the smaller tibial myelinated nerve fibres observed in older adults may reflect axonal atrophy rather than degeneration and regeneration of the myelinated nerve fibres. Further research is needed to investigate the pathologies and molecular mechanisms underlying these age-related morphometric changes and their clinical implications in older adults.

## Teaser

There are few data on peripheral nerve morphology derived from large numbers of older adults. Oveisgharan *et al.* reported morphometric characteristics of 754 247 tibial myelinated nerve fibres from 140 autopsies and their association with age. The study suggests older age associated with smaller myelinated nerve fibres because of axonal atrophy.

## Graphical Abstract

**Graphical Abstract:**
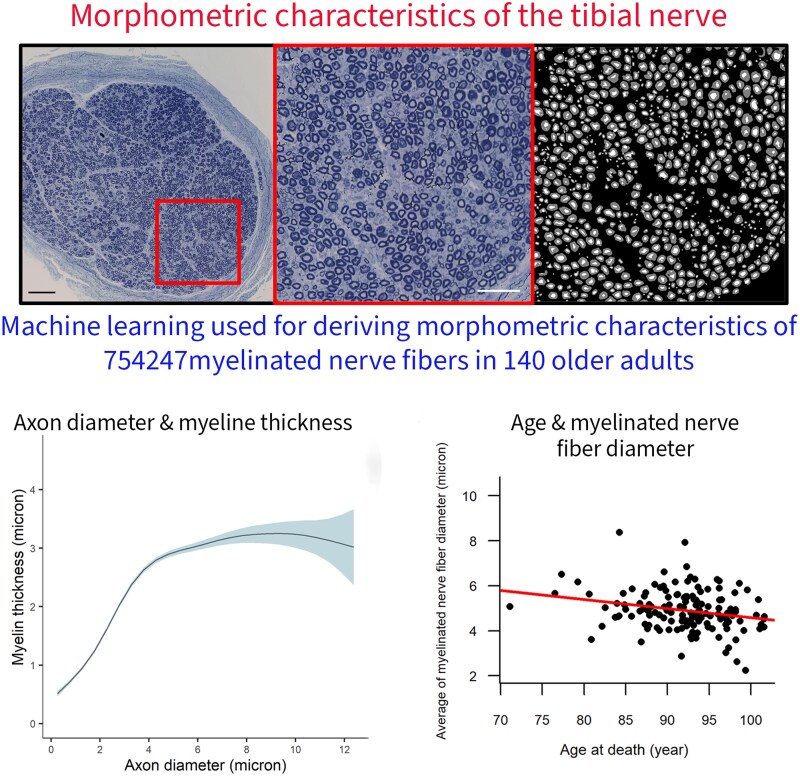

## Introduction

Peripheral nerves consist of motor, sensory and autonomic fibres that connect central control systems to end organs, such as skeletal muscles and sensory receptors. Although clinical electrophysiologic studies show age-related changes in conduction velocity and signal amplitude, few studies have documented the morphological characteristics of peripheral nerves in large numbers of older adults. This knowledge gap impedes research efforts that seek to prevent age-related changes in the musculoskeletal system.

Each myelinated nerve fibre is composed of an axon encircled by myelin. Morphometric characteristics, including myelinated nerve fibre diameter, axon diameter, myelin thickness and *g*-ratio (ratio of axon diameter/myelinated nerve fibre diameter,^1^ which is an index of myelination), provide critical insights into the health of peripheral nerves. There is a paucity of data on the morphometric characteristics of peripheral nerve in older adults, with most available data dating back half a century and drawn either from animal models or from studies that included few adults over 75 years old. Additionally, prior human studies focused primarily on the sural nerve, an exclusively sensory nerve.^1^ Similarly, few studies have examined the relationship between age and morphometric changes in peripheral nerves.^2^ Such studies either examined nerves in aged animals,^3-5^ mostly rats and mice, or were human studies^6-9^ that compared morphometric characteristics of the nerves in older adults with young adults or children. Such earlier work did not focus on age-related changes that occur in later life, when neurodegenerative changes are common. Moreover, published studies reported inconsistent findings about the association between age and morphometric characteristics of the nerves.^6-9^

Myelinated nerve fibres are critical for the rapid transmission of both motor and proprioception impulses. Schwann cells make and are crucial for maintaining the myelin that encircles myelinated axons. Myelination of nerve fibres is made possible via an interaction between axons, Schwann cells and the extracellular matrix.^10,11^ One component of the interaction is the signals that are sent from axons to Schwann cells, such as the neuregulin-1 (NRG1)/erb-b2 receptor tyrosine kinase 3 (ERBB3) pathway.^12^ In fact, the NRG1/ERBB3 pathway is crucial for determining whether an axon is myelinated as well as the degree of myelination. In reverse, Schwann cells release factors including neurotrophins (such as nerve growth factor and brain-derived neurotrophic factor) that upon binding to receptors on the axonal membrane lead to the release of NRG1.^13^ In addition, Schwann cells are crucial for maintaining axons as without supporting Schwann cells axons will retract and degenerate.^14^ Morphometric studies of peripheral nerves are critical to advance our understanding about the complex inter-relationship between axons and Schwann cells by studying the association between axonal diameter and myelin thickness, studying *g*-ratio and studying the molecular mechanisms that underlie the relationship between axons and myelin.^15^ The paucity of morphometric data on large numbers of older adults may account in part for the knowledge gaps about the biology driving degeneration of peripheral nerves in aging adults.

Given that the numbers of adults over 80 years old are growing rapidly, it is imperative to obtain morphometric characteristics of peripheral nerves that include both motor and sensory nerves. Such data are essential for understanding diverse aging phenotypes affected by altered peripheral nerve structure and functions. To fill these knowledge gaps, we quantified morphometric characteristics from postmortem samples of the tibial nerve from participants in the Rush Memory and Aging Project (MAP),^16^ an ongoing community-based clinical–pathological study of chronic conditions of aging. The two primary objectives of the current study were to summarize the morphometric characteristics of the tibial nerve at the time of death and to examine the associations of the morphometric characteristics with age. We examined only the myelinated nerve fibres because they are crucial for mobility that is commonly impaired in aging adults, and because our novel method was not trained or scaled for quantification of unmyelinated axons.

## Methods

### Participants

MAP^16^ began in September 1997 by recruiting older adults living in retirement centres, subsidized housings and personal accommodations across northeastern Illinois. The eligibility criteria were being without known dementia and consenting to annual clinical visits, blood draw and postmortem autopsy that included the tibial nerve. An Institutional Review Board of Rush University Medical Centre approved the study (the approval number is L86121802), and all participants signed informed consent and an Anatomical Gift Act for organ donation. The staff performing the autopsy and collecting the postmortem tissues were blinded to the clinical data collected before death. For the current study, participants with diabetes mellitus were excluded as they might have subclinical diabetic polyneuropathy. The analytic sample consisted of the first 140 consecutive nondiabetic older adults with completed quantification of the morphometric characteristics of the postmortem tibial nerve.

### Tibial nerve preparation and optical image acquisition

The median postmortem interval was 6.8 h (IQR: 5.8–8.5). At autopsy, bilateral tibial nerves were dissected at the popliteal fossa and fixed for histological analysis in a solution containing 4% paraformaldehyde, 1.0% glutaraldehyde in 0.1 M cacodylate buffer at pH 7.4. A segment of the fixed tibial nerve was examined in the current study after its macroscopic blood vessels and adipose tissue were removed using fine forceps under a stereomicroscope.

**Figure 1:**
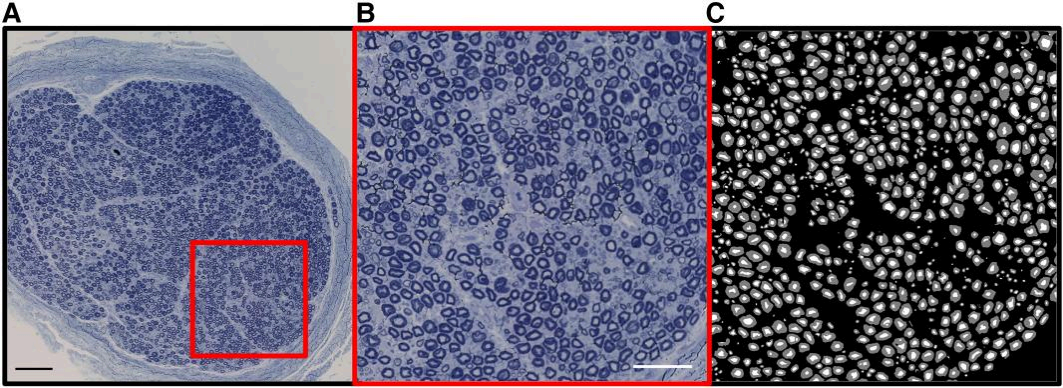
**Segmentation of the myelinated nerve fibres of the tibial nerve obtained from an 84-year old decedent.** Panel **A** shows the overview of the nerve cut in the raw image (×40 objective and ×20 digital magnification with a total magnification of ×800; the scale bar indicates 120 µm); Panel B shows the close-up of the highlighted region of interest (further 6.67 digital magnification with a total magnification of 5333×; scale bar indicates 60 µm); Panel **C** shows the segmentation with the axon in white and the myelin in grey. Measurements are automatically extracted from this mask. For staining, nerve sections on a microscope slide were exposed to 1% Toluidine Blue on a hot plate. When the outer edges of the Toluidine Blue began to dry, the slides were removed from the hot plate and rinsed with deionized water for 30 s each. For tibial nerve preparation and optical image acquisition, see Methods.

A nerve fascicle was carefully dissected while preserving the perineurium and then embedded in Spurr resin.^17,18^ Five microtome thick sections (500 nm) were stained with Toluidine Blue^18^ and mounted on a microscope slide with a coverslip using Permount mounting medium (Fisher Chemical, Pittsburgh, PA). The nerve sections were imaged digitally using an Olympus Slideview VS200 optical microscope (Olympus Corp, Breinigsville, PA) at 40× objective magnification with further 20× or higher digital magnification (Fig. 1). We examined the entire available surface area of the nerve, which was quantified using NDP.view2 software (Hamamatsu, Bridgewater, NJ). The quantified surface area was used for the calculation of myelinated nerve fibres density, which was equal to the number of myelinated nerve fibres/surface area (mm^2^).

### Image analysis

We used a deep learning approach, rather than a traditional image segmentation algorithm, for segmenting axon and myelin because this approach can learn the hidden structure of the tissue by itself, and it is not necessary to hand-select the features.^19^ Segmentations and morphometric analyses were performed using the open-source *AxonDeepSeg* (ADS) software,^19^ and the model was trained using the medical image analysis framework *ivadomed.*^20^ To train the model, images of the nerve sections of three participants were initially used. Two participants were selected based on contrast to reflect the basic features of the entire dataset. A third participant was chosen for its large myelin infoldings and histological alterations representative of other individuals. The manually corrected masks from these three subjects covered more than 4.9 million pixels and included 3560 myelinated nerve fibres. This data was divided into training, validation and testing sets with 75%, 15% and 10% of the whole dataset, respectively. For memory efficiency, images were then divided into 512 × 512 patches with 32-pixel overlaps. The training was performed over 400 epochs (the number of times the entire dataset was passed through the learning algorithm) using a cyclic learning rate and a batch size of six patches. The loss function that led to the best results was a multiclass variation of the traditional Dice loss, which provides an average value over both classes of axon and myelin. Data augmentations were used at train-time including random affine and elastic transforms. Training was performed on a Nvidia Tesla P100-SXM GPU, but the final segmentation inference was done on a regular CPU.

We opted to utilize the U-Net architecture, widely recognized for its effectiveness in biomedical image segmentation. To train the model, we used hyperparameter optimization by applying ∼30 variations of hyperparameters including different network depth (3 or 4), learning rate scheduler (CosineAnnealingLR or CyclicLR), number of epochs (200, 300 or 400), loss function (DiceLoss or MultiClassDiceLoss), and other hyperparameters. The final model was a 2D convolutional U-net of depth 4 with a dropout rate of 25% and a softmax final activation. The size of the model’s input was 512 × 512 with a stride of 480 pixels in both directions. It achieved the best performance on the validation set for the myelin class and was subsequently used to perform the final segmentation of the whole dataset. Evaluated on the held-out testing set, this model achieved a pixel-wise accuracy of 0.9767, a Dice score of 0.8797, a precision of 0.8833 and a recall of 0.8788. This model adequately segments most myelinated nerve fibres for this image domain (Fig. 1). It is publicly available at https://github.com/axondeepseg/model_seg_human_axon-myelin_bf.

Morphological metrics were automatically extracted from the segmentations using *AxonDeepSeg* based on the areas of the segmented axons and myelin. The equivalent diameter of the axons is computed as the diameter of a circle with the same area. Similarly, the equivalent diameter of the whole myelinated nerve fibre is used to estimate myelin thickness. The software can then estimate the *g*-ratio based on these inner and outer diameters. The formulas for calculating the morphometric characteristics were^21^:


$$\mathrm{Axon}\;\mathrm{diameter}=2\sqrt{\left(\frac{axon\;area}{\pi }\right)},$$

$$\mathrm{Myelinated}\;\mathrm{nerve}\;\mathrm{fiber}\;\mathrm{diameter}=2\sqrt{\left(\frac{\left(axon\;\;area+myelin\;\;area\right)}{\pi }\right)},$$

$$g\text{-}\mathrm{ratio}=\frac{axon\;diameter}{myelinated\;nerve\;fiber\;diameter},$$

$$\mathrm{Myelin}\;\mathrm{thickness}=\frac{\left(myelinated\;nerve\;fiber\;diameter-axon\;diameter\right)}{2}.$$

In a prior study, *AxonDeepSeg* showed excellent accuracy in counting myelinated nerve fibres, in segmenting axon and myelin and in calculating axon diameter, myelin thickness and *g*-ratio compared with the gold-standard manual method.^22^ The experimental results can be closely recreated from the training set. Random data augmentation transformations applied to the training data and random initial weights could cause minor variability in performance, but the model could be trained again with the same configuration and achieve almost identical results on the test set. Furthermore, the inference process is deterministic such that segmentation and morphometrics values are perfectly reproducible when using the model weights.

### Age and other covariates

Age at death was calculated using date of birth, obtained from interview at study entry, and the date of death obtained at autopsy. Sex, race/ethnicity and education data were also obtained at study entry.

Diabetes mellitus was present if a participant reported a history of a clinical diagnosis of diabetes mellitus or the use of antidiabetic medications at any visit before death. Histories of four vascular diseases (stroke, myocardial infarction, heart failure and lower extremities claudication) and two vascular risk factors (hypertension and smoking) were also obtained from self-report. A summary variable, number of vascular diseases, was calculated as the number (0 to 4) of the vascular diseases present in a participant.

Body mass index was calculated using measured weight and height. At each annual visit, blood was drawn and examined at Quest Laboratories for measuring creatinine level, which was used for estimation of glomerular filtration rate using the 4-variable Modification of Diet in Renal Disease formula.^23,24^

At each annual visit, 21 neuropsychological tests were administered that were scored by a custom-made algorithm and then reviewed by a neuropsychologist to determine the presence of cognitive impairment. After death, a clinician experienced in the care of dementia reviewed data from all clinical visits and adjudicated the presence of dementia before death according to the established criteria.^25^

### Statistical analysis

The analyses planned for this study were deriving the morphometric characteristics of the myelinated nerve fibres, examining the relationship between axon diameter and myelin thickness, and testing the association between age and the morphometric characteristics. Because the scatter plot of myelin thickness versus axon diameter indicated a nonlinear association, we used a generalized additive mixed model that could accommodate the observed nonlinearity rather than using a planned linear model. Examining the association between age and the distribution of the myelinated nerve fibres by their diameter or between age and *g*-ratio of the myelinated nerve fibres classified by their diameters was also not a planned analysis.

#### Nerve characteristics

We regarded the myelinated nerve fibres as the unit of analysis. We used descriptive statistics to summarize the morphometric characteristics of the myelinated nerve fibres and used the Spearman correlation coefficient to examine correlations between the characteristics. As the scatter plot of the axon diameter and myelin thickness of the myelinated nerve fibres suggested a nonlinear association, we did not use the regular linear regression model to examine the association of axon diameter with myelin thickness. Instead, we used generalized additive mixed models that allow flexible associations between two variables using non-parametric regression while accounting for the correlation between observations by including random effects.^26^ We used the *gamm* function from *mgcv* package^27^ in R, with axon diameter as the model term and myelin thickness as the outcome. A random intercept was included by specifying participant as a random effect, accounting for intra-subject correlation between myelinated nerve fibres within each participant.

#### Age and nerve characteristics

In order to study associations of age-at-death without bias created by the extreme variation in the numbers of myelinated nerve fibres quantified per person, we reduced the data to either person-specific averages or we selected a sample of 100 myelinated nerve fibres at random for each person. The two methods were used in different models. For example, to examine the association between age and the diameter of myelinated nerve fibres, we used person-specific average of the diameter of the myelinated nerve fibres. However, we used 100 randomly selected myelinated nerve fibres for each individual to examine whether age at death was associated with *g*-ratio of small myelinated nerve fibres.

##### Linear regression models

We first calculated the person-specific average of the morphometric characteristics, such as the axon diameter, by determining the mean axon diameter of all myelinated nerve fibres separately for each participant. Then, we used linear regression models to examine the association between age at death and person-specific averages, with sex and other potential confounders included as covariates. Linear regression models were also used to study the association between age at death and myelin nerve fibre density.

##### Compositional data analysis

Next, we used compositional data analysis^28^ to examine the association of age at death with the distribution of myelinated nerve fibre diameters. For each participant, we calculated the percentage of myelinated nerve fibres in each of the six diameter groups (I: 0 to <2, II: 2 to <4, III: 4 to <6, IV: 6 to <8, V: 8 to <10, VI: ≥10 µm). The sum of the percentages for the six groups was 100% in each individual. For example, an average participant had the following distribution of the myelinated nerve fibres: I: 10%, II: 40%, III: 20%, IV: 10%, V: 10%, and VI: 10%, totaling 100%. To be used in a compositional data analysis, one of the categories was the reference (e.g. category I), and five ratios were computed for each participant by dividing the percentage of the myelinated nerve fibres in each of the categories II to VI by the percentage of the myelinated nerve fibres of category I. The five ratios were log-transformed and were examined in relation to age at death. A generalized additive model for location, scale and shape (GAMLSS) was utilized to assess the association between age at death (the model term) and the five log-transformed ratios as the outcome, controlled for sex. The model included both fixed effects and random effects, with a random intercept for each participant. The response variables were modeled using a normal distribution family with default settings for the mean (identity link) and the standard deviation (log link), allowing for heteroscedasticity. The analysis was done using the *gamlss* package in R.^29^

##### Use of GAMLSS model to examine age and g-ratio

We used a similar GAMLSS model to examine the association between age at death and myelinated nerve fibre *g*-ratio. For the latter, we randomly selected 100 myelinated nerve fibres per participant to optimize computational efficiency. In addition to age at death, model covariates included sex, the six diameter groups of the myelinated nerve fibres (with the largest group, ≥10 µm, as the reference), and the five interaction terms between age at death and the myelinated nerve fibre diameter groups. The outcome was the *g*-ratio of myelinated nerve fibres.

Data preparation and linear regression models were done in SAS version 9.4, and generalized additive mixed models, compositional data analyses and the heteroscedastic linear regression analyses were performed in R version 4.2.2. A *P*-value of < 0.05 was considered statistically significant, and no multiple comparison correction was done.

## Results

### Demographic and clinical characteristics

The participants (*N* = 140) had an average age of 90 years at the time of death, and 70% were women. Additional clinical characteristics are summarized in **Table 1**.

**Table 1:** Characteristics of study participants (*N* = 140)

Characteristics	Mean (SD), Median (IQR), *n* (%)
**Demographic**	
Age at death (years), mean (SD)	92.0 (5.4)
Female, *n* (%)	101 (72.1)
White ethnicity	138 (98.6)
Years of education, mean (SD)	15.1 (2.7)
**Clinical at the last visit**	
Hypertension, *n* (%)	94 (67.1)
Smoking history, *n* (%)	50 (35.7)
Body mass index, mean (SD)	25.5 (4.3)
Number of vascular diseases, median (IQR)	1.0 (0.0–1.0)
Stroke, *n* (%)	31 (22.1)
Myocardial infarction, *n* (%)	28 (20.0)
Heart failure, *n* (%)	17 (12.1)
Claudication of lower extremities, *n* (%)	33 (23.6)
Dementia, *n* (%)	59 (42.1)
**Lab**	
Glomerular filtration rate (ml/min/1.73 m^2^), mean (SD)	59.9 (16.0)
Person-specific average of myelinated nerve fibre diameter of tibial nerve, mean (SD) (μm)	4.9 (0.9)
Person-specific average of axon diameter of tibial nerve, mean (SD) (μm)	2.1 (0.4)
Person-specific average of myelin thickness of tibial nerve, mean (SD) (μm)	1.4 (0.3)
Person-specific average of *g*-ratio of tibial nerve, mean (SD)	0.46 (0.04)

### Morphometric characteristics of tibial nerve

A total of 755 287 myelinated nerve fibres were examined (Supplementary Fig. 1). Of those, 1040 myelinated nerve fibres were excluded because of invalid estimated numbers, such as negative myelin thickness or *g*-ratio greater than 1. Therefore, 754 247 myelinated nerve fibres were included in the further analyses, with an average of 5387 myelinated nerve fibres per participant (SD = 3,436, range: 299 to 19 892).

**Figure 2:**
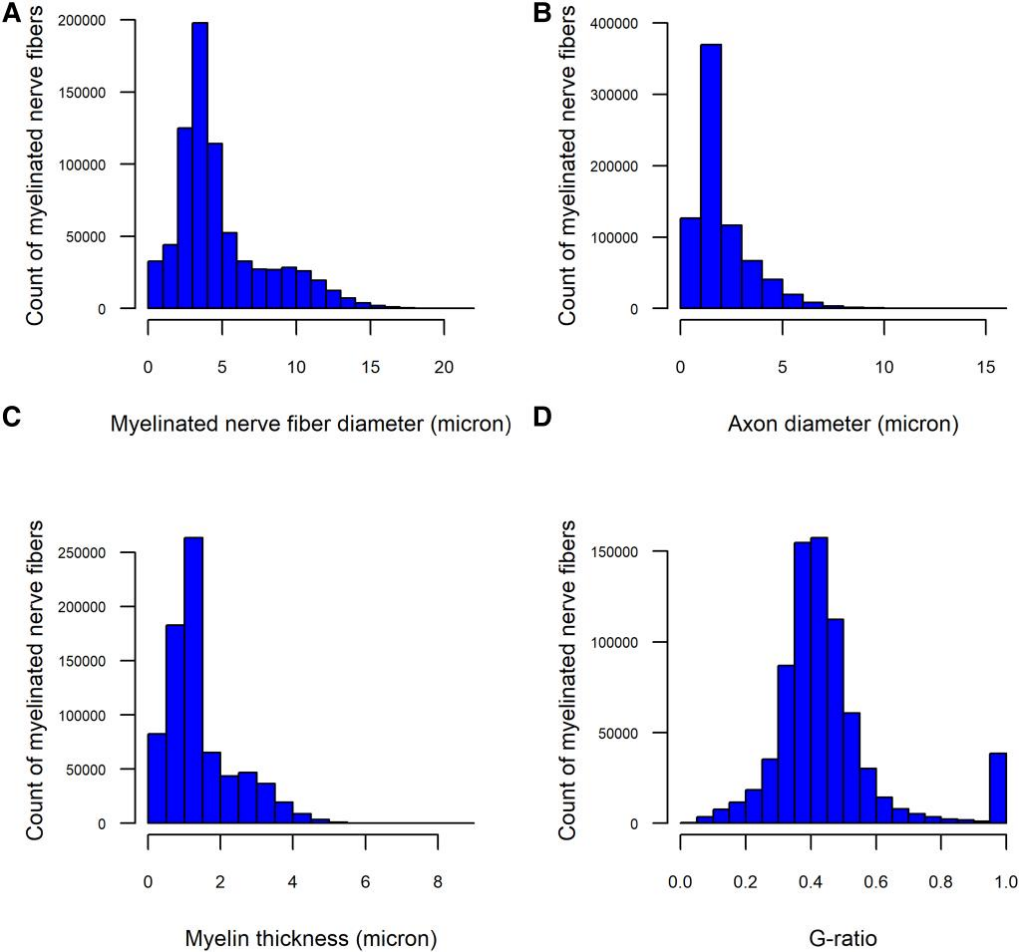
**Distribution of morphometric characteristics of tibial myelinated nerve fibres from older decedents.** Histogram of 754 247 myelinated nerve fibres obtained at autopsy from the tibial nerves of 140 older decedents illustrating their distribution by the diameter of the myelinated nerve fibre (**A**), axonal diameter (**B**), myelin thickness (**C**), and *g*-ratio (**D**). For example, the panel **A** illustrates that most of the myelinated nerve fibres were 3–5 µm in diameter, and there is a unimodal, not a bimodal, distribution of the myelinated nerve fibres by their diameter.

The average diameter of myelinated nerve fibres was 4.9 µm (SD = 3.1, range: 0.26–21.9), and the average diameter of axons was 2.0 µm (SD = 1.4, range: 0.26–15.6). Figure 2 shows the distributions of the diameters of myelinated nerve fibres and axons. Most myelinated nerve fibres measured between 3 and 5 µm, and the majority of axons had a diameter near 2 µm. Contrary to previous findings, we did not observe a bimodal distribution of myelinated nerve fibre diameters.^6^

**Figure 3:**
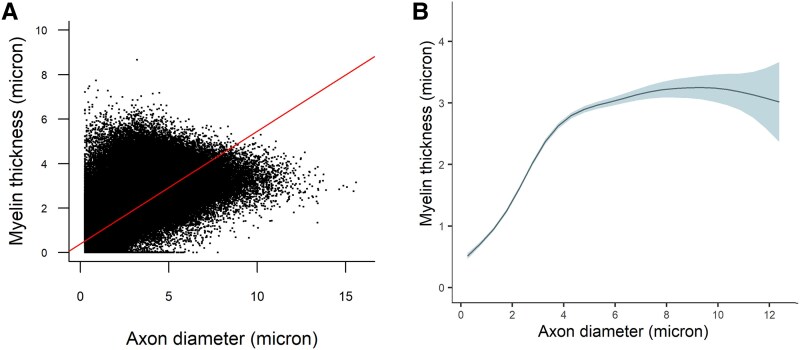
**Associations of axon diameter with myelin thickness in tibial myelinated nerve fibres from older decedents.** Scatter plot of the 754 247 myelinated nerve fibres, obtained from the tibial nerves of 140 older decedents, illustrating the association between axon diameter and myelin thickness is shown in **A**. Each dot represents one myelinated nerve fibre. The included line derived from a least-square linear regression model (myelin thickness (µm) = 0.393 + 0.506 × axon diameter(µm)) shows that the regression line does not fit the data when large axons are considered, and the scatter plot suggests that a nonlinear model should be examined between the two variables. **B** shows the association between axon diameter and myelin thickness derived from a generalized additive mixed model, using the *gamm* function from *mgcv* package^27^ in **R**, that allows flexible associations between two variables by using non-parametric regression while accounting for the correlation between observations by using random effects.^26^ The analysis was performed on 14 000 myelinated nerve fibres, 100 randomly selected myelinated nerve fibres from 140 participants. The *y*-axis is the estimated myelin thickness, and the *x*-axis is the axon diameter. The curve, derived from the generalized additive mixed model, illustrates the nonlinear association between axon diameter and myelin thickness: myelin gets thicker as axon diameter increases until 8 µm, above which myelin thickness is not correlated with axon diameter. The shaded area around the curve indicates 95% confidence interval of myelin thickness.

The average myelin thickness was 1.4 µm (SD = 0.96, range: 0.0–8.7). We examined the relationship between axon diameter and myelination thickness.^1^ On average, larger axons had thicker myelin (*ρ* = 0.73, *P* < 0.001). However, inspection of the scatter plot (Fig. 3A) revealed a nonlinear relationship between them. To model this nonlinear association, we randomly selected 100 myelinated nerve fibres per participant and applied generalized additive mixed models. The analysis confirmed a nonlinear relationship (Fig. 3B), with an estimated effective degrees of freedom of 7.5 (*P* < 0.001), compared to a linear model’s single degree of freedom. Axon diameter explained 57.4% of the variance in myelin thickness. Myelin thickness increased with axon diameter up to 4 µm at a rate of 0.5 µm per 1-µm increase in axon diameter. Between 4 and 8 µm, the rate of increase slowed to 0.125 µm per 1 µm increase in axon diameter. For axons >8 µm, myelin thickness plateaued and did not increase further.

Prior studies have suggested that a *g*-ratio between 0.6 and 0.7 is optimal for the fastest conduction of action potentials.^1^ As illustrated in Fig. 2, the average *g*-ratio was low (mean = 0.45, SD = 0.17, range: 0.02–1), with only 3.0% (*n* = 22 483) of myelinated nerve fibres falling within the optimal *g*-ratio range.

### Age and myelinated nerve fibre diameter

**Figure 4:**
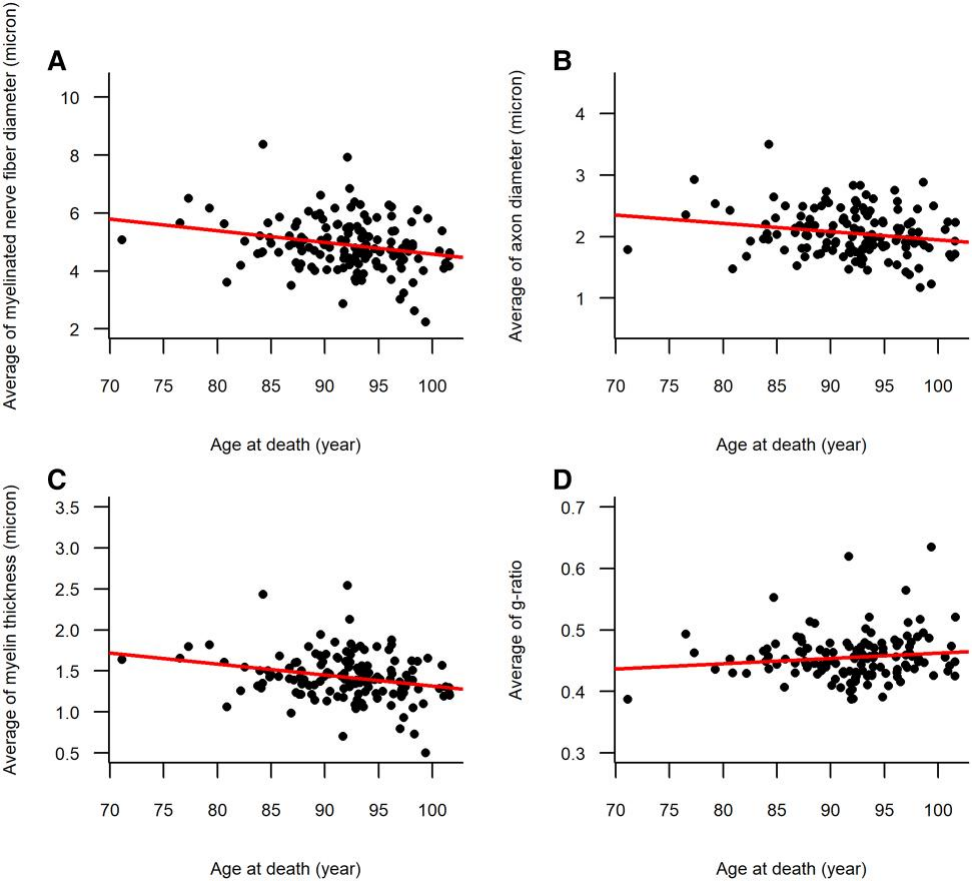
**Scatter plots of age at death in relation to person-specific averages of myelinated nerve fibre characteristics of tibial nerve.** The four scatter plots, including data of 140 participants, illustrate the association between age at death and person-specific averages of myelinated nerve fibres diameter (**A**), axonal diameter (**B**), myelin thickness (**C**) and *g*-ratio (**D**). Each dot represents a participant’s age at death (*x*-axis) and one of the person-specific averages of the morphometric characteristics calculated over a mean of 5387 (SD = 3436) tibial myelinated nerve fibres per participant. The included lines are derived from the least-square linear regression models (**A**: person-specific average of myelinated nerve fibres diameter (µm) = 8.588 + (−0.040) × age-at-death (year); **B**: person-specific average of axon diameter (µm) = 3.291 + (−0.013) × age-at-death (year); **C**: person-specific average of myelin thickness (µm) = 2.648 + (−0.013) × age-at-death (year); **D**: person-specific average of *g*-ratio = 0.376 + (0.001) × age-at-death (year)). The regression lines indicate that older age at death was associated with smaller myelinated nerve fibres shown in **A**, smaller axons in **B**, thinner myelin in **C**, but not associated with *g*-ratio in **D**.

We examined the relationship between age at death and the person-specific average for each morphometric nerve characteristic. Older age at death was associated with smaller myelinated nerve fibres (*ρ* = −0.22, *P* = 0.008) (Fig. 4). In a linear regression model controlling for sex, age at death was related to myelinated nerve fibre diameter (estimate = −0.041, SE = 0.014, *P* = 0.004). Based on estimates from the model, we calculated the effect size of age at death (Supplementary Table 1). The model’s intercept was 4.9, and the estimate of the association between age and the person-specific average of diameter of myelinated nerve fibres was −0.04. The above numbers indicate that the average of diameters of myelinated nerve fibres in an average 92-year-old individual was 4.9 µm and in a 97-year-old individual was 0.02 µm (5*-0.04) or 4% less, equal to 4.7 µm. The association persisted after adjusting for vascular risk factors and diseases (Table 2). Furthermore, we examined whether sex modified the association between age at death and myelinated nerve fibre diameter by adding an interaction term between age at death and sex to the linear regression model. The interaction term was not significant (estimate = 0.059, SE = 0.035, *P* = 0.093), indicating the association between age at death and myelinated nerve fibre diameter did not differ by sex.

**Table 2:** Association of age at death with the person-specific averages of the morphometric characteristics of tibial nerve controlled for sex and other characteristics

Model terms	Estimate (SE), *P*-value			
	Myelinated axon diameter	Axon diameter	Myelin thickness	*g*-ratio
Ref: Age at death	−0.041 (0.014), 0.004	−0.014 (0.006), 0.017	−0.013 (0.004), 0.003	0.001 (0.001), 0.185
Model 1: Ref. + hypertension	−0.039 (0.014), 0.007	−0.013 (0.006), 0.032	−0.013 (0.005), 0.004	0.001 (0.001), 0.174
Model 2: Model 1 + smoking	−0.039 (0.014), 0.008	−0.013 (0.006), 0.034	−0.013 (0.005), 0.005	0.001 (0.001), 0.188
Model 3: Model 2 + Body mass index	−0.039 (0.014), 0.008	−0.013 (0.006), 0.036	−0.013 (0.005), 0.005	0.001 (0.001), 0.177
Model 4: Model 3 + Vascular diseases	−0.038 (0.014), 0.009	−0.013 (0.006), 0.039	−0.013 (0.005), 0.005	0.001 (0.001), 0.185
Model 5: Model 4 + glomerular filtration rate	−0.039 (0.014), 0.007	−0.014 (0.006), 0.017	−0.013 (0.004), 0.003	0.001 (0.001), 0.185

The cells’ entries are derived from 24 separate linear regression models. For each model, the terms included are described in the left column and the outcome is one of the person-specific morphometric characteristics shown at the head of the columns 2 through 5. All the models were controlled for sex.

We next examined whether the association between older age at death and smaller myelinated nerve fibres was because of smaller axons or less myelination. In three separate linear regressions with person-specific averages of axon diameter, myelin thickness and *g*-ratio as the outcomes, older age at death was associated with both smaller axons and less myelination but not with *g*-ratio (Table 2; Fig. 4). These associations remained consistent after controlling for vascular risk factors and diseases (Table 2).

### Age and the distribution of myelinated nerve fibres by diameter groups

In the prior analyses, older age was associated with a lower mean of the person-specific average of myelinated nerve fibre diameter. However, as illustrated in Fig. 2, myelinated nerve fibre diameters do not follow a uniform distribution. Therefore, we examined whether age at death was associated with distribution of myelinated nerve fibre diameters. We categorized myelinated nerve fibres into six diameter groups, 0 to <2, 2 to <4, 4 to <6, 6 to <8, 8 to <10, and 10 µm or larger (Supplementary Table 2). We used compositional data analysis to assess the association of age at death with the distribution of myelinated nerve fibres across the diameter groups. Our analysis indicated that older age at death was associated with a lower percentage of myelinated nerve fibres in the groups >8 µm (Table 3): [Age × (percent of myelinated nerve fibres 8 to <10 µm/percent of myelinated nerve fibres <2 µm): estimate = −0.035, SE = 0.013, *P* = 0.008; Age ×  (percent of myelinated nerve fibres ≥10 µm/percent of myelinated nerve fibres <2 µm): estimate = −0.037, SE = 0.016, *P* = 0.019]. These significant interaction terms had negative estimates indicating that older age was associated with lower percentage of the myelinated nerve fibres in the 8 to <10 µm or ≥10 µm category.

**Table 3:** Association of age at death with the percentage of tibial myelinated nerve fibres of different diameters

Model terms	Estimate (SE), *P*-value
Intercept	1.313 (0.042), <0.001
Age at death	0.001 (0.007), 0.862
Sex	0.016 (0.056), 0.771
Percent of myelinated nerve fibres 2 to <4 µm/percent of myelinated nerve fibres <2 µm	REF.
Percent of myelinated nerve fibres 4 to <6 µm/percent of myelinated nerve fibres <2 µm	−0.659 (0.070), <0.001
Percent of myelinated nerve fibres 6 to <8 µm/percent of myelinated nerve fibres <2 µm	−1.605 (0.070), <0.001
Percent of myelinated nerve fibres 8 to <10 µm/percent of myelinated nerve fibres <2 µm	−1.758 (0.080), <0.001
Percent of myelinated nerve fibres ≥10 µm/Percent of myelinated nerve fibres <2µm	−1.618 (0.095), < 0.001
Age × (percent of myelinated nerve fibres 4 to <6 µm/percent of myelinated nerve fibres <2 µm)	−0.007 (0.012), 0.573
Age × (percent of myelinated nerve fibres 6 to <8 µm/percent of myelinated nerve fibres <2 µm)	−0.018 (0.012), 0.130
Age × (percent of myelinated nerve fibres 8 to <10 µm/percent of myelinated nerve fibres <2 µm)	−0.035 (0.013), 0.008
Age × (percent of myelinated nerve fibres ≥10 µm/percent of myelinated nerve fibres <2 µm)	−0.037 (0.016), 0.019

The second column numbers are derived from a heteroscedastic normal distribution log-ratio regression model with the model terms listed in the left column and the outcome is ratio of the percentage of myelinated nerve fibres in 2 to <4, 4 to <6, 6 to <8, 8 to <10, or ≥10 µm to percentage of myelinated nerve fibres with the size of 0 to <2 µm.

Examination of the dispersion submodel indicated that age at death was not associated with higher or lower variance in a specific group of myelinated nerve fibre diameter because the interaction terms between age at death and diameter groups were not significant (Supplementary Table 3). However, when we removed the interaction terms, older age at death was associated with more variance (estimate = 0.019, SE = 0.004, *P* < 0.001) in the percentages of the myelinated nerve fibres across the six diameter groups (Supplementary Table 4), indicating greater variability in the myelinated nerve fibres size with aging.

### Age and myelinated nerve fibres density

In prior analyses, older age at death was associated with a smaller person-specific average of myelinated nerve fibre diameter, primarily due to lower percentages of large-diameter myelinated nerve fibres (>8 µm). The lower percentages in large myelinated nerve fibres could be attributed to either the loss of large myelinated nerve fibres or their atrophy, resulting in smaller myelinated nerve fibres. To indirectly distinguish the two possibilities, we examined the association of age at death with myelinated nerve fibre density. Myelinated nerve fibre density had a mean of 4993.4 count/mm^2^ (SD = 2076.2, range: 594.1–9898.7) and was not associated with age at death (Spearman *ρ* = 0.06, *P* = 0.460). Moreover, in a linear regression model controlling for sex, age at death was not associated (estimate = 3.1, SE = 34.6, *P* = 0.928) with myelinated nerve fibre density (Supplementary Fig. 2). These findings suggest that the association of age at death with smaller myelinated nerve fibres is more likely due to the atrophy of large myelinated nerve fibres rather than their loss.

### Age and *g*-ratio of myelinated nerve fibre diameter groups

One hypothesis explaining the association between older age and smaller myelinated nerve fibres is the accumulation of regenerated myelinated nerve fibres, which are characterized by small sizes, thin myelin, and high *g*-ratios. If this hypothesis holds true, with older age at death, we would expect to see higher *g*-ratios in small-diameter axon groups. We used a heteroscedastic linear regression model to examine the relation between age at death and *g*-ratio of myelinated nerve fibres, categorized by diameter, using 100 randomly selected myelinated nerve fibres per participant. The results (**Table 4**) indicated that, although the small-diameter group (myelinated nerve fibre diameter <2 µm) had larger mean *g*-ratio than the larger-diameter groups, the association of older age at death with *g*-ratio in this group was in the opposite direction (estimate = −0.003, SE = 0.001, *P* = 0.035). For example, for every 5 years older age at death, the average *g*-ratio in the <2 µm group was lower by 0.015. Furthermore, age at death was not related to the *g*-ratio in other small-diameter groups (2–4 and 4–6 µm). These findings suggest that the association between older age at death and smaller myelinated nerve fibres is not due to the accumulation of regenerated small myelinated nerve fibres with thinner myelin and larger *g*-ratio.

**Table 4:** Association of age at death with *g*-ratio of tibial myelinated nerve fibres classified by their diameter

Model terms	Estimate (SE), *P*-value
Intercept	0.429 (0.003), <0.001
Age at death	0.000 (0.001), 0.634
Sex	−0.009 (0.002), <0.001
Myelinated nerve fibres <2µm	0.358 (0.007), <0.001
Myelinated nerve fibres 2 to <4 µm	−0.017 (0.003), <0.001
Myelinated nerve fibres 4 to <6 µm	−0.011 (0.003), <0.001
Myelinated nerve fibres 6 to <8 µm	−0.015 (0.004), <0.001
Myelinated nerve fibres 8 to <10 µm	−0.019 (0.004), <0.001
Myelinated nerve fibres ≥10 µm	REF
Age × Myelinated nerve fibres <2 µm	−0.003 (0.001), 0.035
Age × Myelinated nerve fibres 2 to <4 µm	−0.001 (0.001), 0.130
Age × Myelinated nerve fibres 4 to <6µ m	−0.000 (0.001), 0.760
Age × Myelinated nerve fibres 6 to <8 µm	0.001 (0.001), 0.097
Age × Myelinated nerve fibres 8 to <10 µm	0.002 (0.001), 0.028

The second column data are derived from a heteroscedastic linear regression model for the outcome *g*-ratio with the model terms listed in the left column. To account for the correlation between the 100 randomly selected tibial myelinated nerve fibres from each participant that were used in the analysis, a random effect for participant was included in the model. The analysis indicates that in the target myelinated nerve fibre group with a diameter <2 µm, older age was not associated with a higher *g*-ratio, an indirect evidence of more regenerated myelinated nerve fibres.

### Sensitivity analyses

Although the image analysis software was trained for the segmentation and quantification of myelinated nerve fibres, the calculated *g*-ratio derived from the software’s measurements was equal to one in 5.0% (*n* = 37 729) of the myelinated nerve fibres, an indication of no myelin, possibly because of thin myelin, errors caused during tissue preparation, or software errors. In a series of sensitivity analyses, we excluded the myelinated nerve fibres with *g*-ratio = 1 and repeated the analyses. The main findings did not change (Supplementary Tables 5–8,Supplementary Figs 3–6): the average diameter of the myelinated nerve fibres was 5.2 µm (SD = 0.9), the average *g*-ratio was 0.42 (SD = 0.02), axon diameter and myelin thickness showed a nonlinear correlation, age at death was related to smaller myelinated nerve fibres but was not related to *g*-ratio or myelinated nerve fibre density. Moreover, older age at death was associated with a smaller fraction of large-diameter myelinated nerve fibres (>8 µm) but not associated with higher *g*-ratio at small-diameter myelinated nerve fibres, an indirect measure of regenerated myelinated nerve fibres. Similarly, in separate series of sensitivity analyses we removed myelinated nerve fibres with a diameter < 1 µm (*n* = 32 707) or an axonal diameter < 0.8 µm (*n* = 66 550), which are theoretically more representative of unmyelinated rather than myelinated nerve fibres. Excluding these small nerve fibres from our analysis did not change the study’s main findings (Supplementary Tables 9–16; Supplementary Figs 7–14).

## Discussion

We employed machine learning methods to quantify morphometric characteristics for more than 750 000 myelinated nerve fibres from postmortem tibial nerves, obtained from 140 deceased community-dwelling older adults participating in a longitudinal clinical-autopsy cohort study. Consistent with previous research, we observed a wide range of myelinated nerve fibre diameters, from <2 µm to >10 µm. A nonlinear association between axon diameter and myelin thickness emerged, with myelin thickness increasing along axon diameter up to 8 µm, beyond which it plateaued. As expected, older age was associated with smaller myelinated nerve fibres, smaller axons, and thinner myelin. However, the *g*-ratio, a measure used to assess axonal myelination, was not associated with age. Further analyses suggested that the association of older age with smaller myelinated nerve fibres was primarily driven by a relative loss of myelinated nerve fibres >8 µm, rather than by increased regeneration of smaller myelinated nerve fibres. Future studies are needed to uncover the underlying pathologies and molecular mechanisms of these morphologic findings and their potential links to physical function impairment in older adults.

Though impaired mobility is common in older adults and loss of ambulation ranks as the second most feared consequence of aging after dementia, the age-related morphologic changes in peripheral nerves of older adults have been understudied. Although nerve biopsies can be obtained from older adults, the biopsies have typically focused on the sural sensory nerve whose biopsy does not impair clinical function except for limited distal sensory loss in the foot. Nonetheless, due to the invasiveness and side effects associated with sural nerve biopsies,^30^ few studies include large numbers of older adults across the full health spectrum since sicker and frail older adults are generally unable to participate in studies requiring travel to specialized labs or tertiary medical centres. While harvesting postmortem nerves can circumvent some of these limitations, few autopsy series collect mixed peripheral nerves at the time of autopsy as specialized expertise is required for processing and assessing peripheral nerves.

Previous studies examining the morphometric characteristics of human peripheral nerves have generally been small (*n* ≤ 35) and included few (*n* ≤ 5) adults older than 75 years.^6-9^ Moreover, most studies have focused on the sural nerve, highlighting the knowledge gap about nerves linking the CNS with motor-related phenotypes. The current study extends prior studies by providing morphometric characteristics of a mixed-peripheral nerve in 140 older adults. Additionally, by leveraging advanced technologies and statistical methods, we were able to sample many myelinated nerve fibres from each peripheral nerve and provide new insights into the relationship between axon diameter and myelin thickness, as well as the association between age and the morphometric characteristics of the myelinated nerve fibres.

The mean myelinated nerve fibre diameter in our study was 4.9 µm, and we found that older age was associated with smaller myelinated nerve fibre diameter. A prior study reported a larger mean myelinated nerve fibre diameter (6.3 µm).^8^ However, that study focused on a sensory nerve in the upper extremity and included no participant over the age of 60. Several mechanisms have been proposed to explain age-related atrophy of myelinated nerve fibre diameter. One hypothesis focuses on the increased regeneration of myelinated nerve fibres to compensate for the age-related loss of anterior horn cells and dorsal root ganglion cells.^2,9^ Yet, compensatory regenerated myelinated nerve fibres are small, with thin myelin and a high *g*-ratio.^31^ Contrary to this hypothesis, our analysis showed that older age was not associated with either the average *g*-ratio or a higher *g*-ratio in small myelinated nerve fibres. These findings suggest that age-related reduction in myelinated nerve fibre size may result from processes other than axonal regeneration, potentially reflecting axonal shrinkage without disruption of the motor or sensory cell bodies, similar to the reduction in muscle fibre area in disuse. Smaller myelinated nerve fibres and smaller axons may be due to loss of neurofilaments,^32^ the major determinants of axonal diameter,^33^ decline in nerve trophic factors such as nerve growth factor receptors,^32^ slower axonal transport^34^ that provide nutrients by anterograde transport from soma to axons and provide trophic factors to soma by retrograde transport, lower nerve perfusion because of more vascular resistance with advancing age,^35^ degeneration of target organs (such as muscle fibres^36^ or dermal sensory organs^37^) that provide crucial trophic factors that maintain structural elements of the peripheral nerve, or inadequate activation of repair signals in aged Schwann cells.^38^ The sympathetic branch of the autonomic nervous system innervates the vasa nervorum, which is essential for maintaining the structure and function of myelinated nerve fibres. Age-dependent deficits in sympathetic innervation have been reported to lead to sarcopenia as a consequence of alterations in the function of myelinated motor axons.^39-42^

Most probably, the mechanisms described above act gradually over years and may manifest linear or nonlinear^43^ effects on longitudinal trajectories of the morphometric characteristics of the nerves. In addition, there should be resilience factors^44^ against these age-related mechanisms affecting myelinated nerve fibres calibre. In fact, the observed age-related axonal atrophy may be due to degenerative changes exceeding the buffering capacity of resilience factors.^45^ As examples, education^46^ and early-life cognitive enrichment^47^ are resilience factors in aging-related cognitive decline caused by Alzheimer’s disease pathology. Yet, even adults with many years of education develop dementia when the burden of Alzheimer’s disease pathology exceeds the resilience capacity of education. Similarly, modifiable life style factors such as physical activity may provide resilience against age-related axonal atrophy. Therefore, the current findings need to be replicated in more diverse populations of older adults and complemented with longitudinal *in vivo* data collection. This approach can elucidate the time course, biology and reversibility of changes in the morphology of peripheral nerves in old age.

We found that the major group of myelinated nerve fibres whose numbers decreased with advancing age was those >8 µm. This was also confirmed by the absence of the bimodal distribution of myelinated nerve fibre diameters reported in prior studies.^6^ Since larger myelinated nerve fibres conduct nerve impulses faster, the shrinkage in larger myelinated nerve fibres may partially explain the slower nerve conduction velocity in older adults. It is possible that, due to their size, larger myelinated nerve fibres are more vulnerable to reduced blood supply and mitochondrial energy production that occur with advancing age.^48,49^

The thickness of peripheral nerve myelin has been the focus of many prior reports because of the crucial role myelin plays in the bioelectric characteristics of peripheral nerves. Prior studies have reported inconsistent associations between axon diameter and myelin thickness.^1^ An important finding in the current study was the nonlinear relationship between axon diameter and myelin thickness, with thicker myelin observed in larger axons though the association plateaued after an axon diameter of 8 µm. The observation of thicker myelin in larger axons is congruent with the studies reporting continuous communication between axons and Schwann cells.^10^ However, it may be possible that extra thick myelin is not compatible with viable axons, considering the dependency of axonal maintenance on insulating Schwann cells.^50^ As myelin thickness increases, the Schwann cell nucleus gets farther from the axon, which limits the maximum thickness of myelin in very large axons.

The average *g*-ratio in the current study was 0.45, smaller than reported in other studies.^7,8^ In the current study the participants were older adults with a mean age of death older than 90 years and did not include younger adults who composed the participants of the studies that reported higher *g*-ratio for the peripheral nerves. In fact, in a seminal study with 27 participants with three older than 70 years *g*-ratio of the myelinated nerve fibres of the oldest 77-year-old participant was on average lower than younger participants.^7^ We hypothesize that aging affects axonal diameter more than myelin thickness, which can explain lower *g*-ratio in the current study, and the reason of not finding an association between age and *g*-ratio in the current study was inadequate power because of not including participants from midlife. Besides disproportionate atrophy of axons due to aging, another possibility for low *g*-ratio is aging-related adaptive myelination,^51,52^ as reported in the central nervous system and may also coexist in the peripheral nervous system. Further studies of mixed-nerves in a larger number of adults across the lifespan will be needed to draw definitive inferences about the *g*-ratio reported in our study. In addition, it is unclear whether the differences between the current study and prior studies are due to the specific nerve examined or the age range studied.^53^ Nonetheless, only 3% of the myelinated nerve fibres had a *g*-ratio between 0.6 and 0.7 that is optimal for fast nerve conduction velocity necessary for superlative human behavior.^1^ This may partially explain the loss of sensorimotor function with advancing age.

Density of myelinated nerve fibres was not related to age in the current study. However, in two smaller autopsy studies of older adults being older at death was associated with a less density of myelinated nerve fibres of the tibial nerve.^54,55^ Several differences in the methods of data collection and analyses may underlie this discrepancy including sample size (140 versus 13), postmortem interval, width of the examined nerve area, and exclusion of patients with diabetes and use of machine learning for segmentation of myelinated nerve fibres in the current study. Nonetheless, the discrepancy underscores the need for replication of the current study findings in other *in vivo* and *ex vivo* studies.

Several limitations must be noted. In the current study, we did not examine unmyelinated axons, which are the major axons for conducting nociception or autonomic innervation. We hope to develop the necessary methods and algorithms to examine unmyelinated axons in these samples in future studies. Additionally, we did not quantify some reported qualitative changes of myelinated nerve fibres, such as myelin infolding, wrinkling and myelin loops,^5^ or the presence of glycogen vacuoles or Reich granules.^56^ The sample was selected from participants willing to undergo autopsy and consisted almost entirely of whites mostly in eight to ninth decades of life with more years of education than average. Further studies in more diverse samples with a wider age range will be needed to confirm the generalizability of the findings. The morphometric characteristics were calculated using machine learning, not the gold standard for these types of studies, which inherently includes some approximation in the segmentation and quantification of the myelinated nerve fibres as was evidenced by 5% of the myelinated nerve fibres whose *g*-ratio was quantified to be equal to one. However, exclusion of the myelinated nerve fibres with *g*-ratio equal to 1 or exclusion of the small myelinated nerve fibres (with a diameter < 1 µm or axon diameter < 0.8 µm) as a proxy of structures other than myelinated nerve fibres did not change the results. Moreover, our validation study showed that our approach had a 98% accuracy compared with the manual pathological collection of indices. Several strengths support the study findings including large number of sample size, advanced statistical methods and short postmortem interval.

## Supplementary Material

fcaf267_Supplementary_Data

## Data Availability

The data including the raw images of the nerve sections are available for sharing through Rush Alzheimer’s Disease Research Centre Resource Sharing Hub at www.radc.rush.edu. To obtain data, the researchers should fill a brief proposal describing the scientific question, the background and approach, and sign a data use agreement.

## References

[1] Thomas P, Ochoa J. Microscopic anatomy of the peripheral nervous system. Peripheral neuropathy. 2nd ed. WB Saunders Company; 1984:39–96.

[2] Thomas PK . Electrophysiological and morphological changes in the peripheral nervous system with ageing. Electroencephalogr Clin Neurophysiol Suppl. 1999;50:103–108. 10689451

[3] Jeronimo A, Jeronimo CAD, Rodrigues Filho OA, Sanada LS, Fazan VPS. A morphometric study on the longitudinal and lateral symmetry of the sural nerve in mature and aging female rats. Brain Res. 2008;1222:51–60. 18585691 10.1016/j.brainres.2008.05.055

[4] Soltanpour N, Asghari Vostacolaee Y, Pourghasem M. Comparison of morphometric aspects of light and electron microscopy of the hypoglossal nerve between young and aged male wistar rats. Cell J. 2012;13(4):229–236. 23508137 PMC3584479

[5] Chase MH, Engelhardt JK, Adinolfi AM, Chirwa SS. Age-dependent changes in cat masseter nerve: An electrophysiological and morphological study. Brain Res. 1992;586(2):279–288. 1521161 10.1016/0006-8993(92)91637-t

[6] O’Sullivan DJ, Swallow M. The fibre size and content of the radial and sural nerves. J Neurol Neurosurg Psychiatry. 1968;31(5):464–470. 4303798 10.1136/jnnp.31.5.464 PMC496402

[7] Jacobs JM, Love S. Qualitative and quantitative morphology of human sural nerve at different ages. Brain J Neurol. 1985;108(Pt 4):897–924. 10.1093/brain/108.4.897 4075078

[8] Chentanez V, Agthong S, Huanmanop T, Pairoh S, Kaewsema A. Morphometric analysis of the human superficial radial nerve. Anat Sci Int. 2010;85(3):167–170. 20127432 10.1007/s12565-010-0073-7

[9] Ugrenović S, Jovanović I, Vasović L, Kundalić B, Čukuranović R, Stefanović V. Morphometric analysis of the diameter and g-ratio of the myelinated nerve fibers of the human sciatic nerve during the aging process. Anat Sci Int. 2016;91(3):238–245. 25976073 10.1007/s12565-015-0287-9

[10] Taveggia C . Schwann cells-axon interaction in myelination. Curr Opin Neurobiol. 2016;39:24–29. 27089429 10.1016/j.conb.2016.03.006

[11] Wilson ER, Della-Flora Nunes G, Weaver MR, Frick LR, Feltri ML. Schwann cell interactions during the development of the peripheral nervous system. Dev Neurobiol. 2021;81(5):464–489. 32281247 10.1002/dneu.22744 PMC7554194

[12] Nave KA, Salzer JL. Axonal regulation of myelination by neuregulin 1. Curr Opin Neurobiol. 2006;16(5):492–500. 16962312 10.1016/j.conb.2006.08.008

[13] Esper RM, Loeb JA. Neurotrophins induce neuregulin release through protein kinase Cdelta activation. J Biol Chem. 2009;284(39):26251–26260. 19648576 10.1074/jbc.M109.002915 PMC2785313

[14] Britsch S, Goerich DE, Riethmacher D, et al The transcription factor Sox10 is a key regulator of peripheral glial development. Genes Dev. 2001;15(1):66–78. 11156606 10.1101/gad.186601 PMC312607

[15] Wolbert J, Li X, Heming M, et al Redefining the heterogeneity of peripheral nerve cells in health and autoimmunity. Proc Natl Acad Sci U S A. 2020;117(17):9466–9476. 32295886 10.1073/pnas.1912139117 PMC7196786

[16] Bennett DA, Schneider JA, Buchman AS, Barnes LL, Boyle PA, Wilson RS. Overview and findings from the rush memory and aging project. Curr Alzheimer Res. 2012;9(6):646–663. 22471867 10.2174/156720512801322663 PMC3439198

[17] Spurr AR . A low-viscosity epoxy resin embedding medium for electron microscopy. J Ultrastruct Res. 1969;26(1):31–43. 4887011 10.1016/s0022-5320(69)90033-1

[18] Hayat M . Principles and techniques of electron microscopy: Biological applications. 4th ed. Cambridge University Press; 2000.

[19] Zaimi A, Wabartha M, Herman V, Antonsanti PL, Perone CS, Cohen-Adad J. AxonDeepSeg: Automatic axon and myelin segmentation from microscopy data using convolutional neural networks. Sci Rep. 2018;8(1):3816. 29491478 10.1038/s41598-018-22181-4 PMC5830647

[20] Gros C, Lemay A, Cohen-Adad J. SoftSeg: Advantages of soft versus binary training for image segmentation. Med Image Anal. 2021;71:102038. 33784599 10.1016/j.media.2021.102038

[21] Duval T, Saliani A, Nami H, et al Axons morphometry in the human spinal cord. NeuroImage. 2019;185:119–128. 30326296 10.1016/j.neuroimage.2018.10.033

[22] Daeschler SC, Bourget MH, Derakhshan D, et al Rapid, automated nerve histomorphometry through open-source artificial intelligence. Sci Rep. 2022;12(1):5975. 35396530 10.1038/s41598-022-10066-6 PMC8993871

[23] Buchman AS, Tanne D, Boyle PA, Shah RC, Leurgans SE, Bennett DA. Kidney function is associated with the rate of cognitive decline in the elderly. Neurology. 2009;73(12):920–927. 19657107 10.1212/WNL.0b013e3181b72629 PMC2754333

[24] Brosius FC, Hostetter TH, Kelepouris E, et al Detection of chronic kidney disease in patients with or at increased risk of cardiovascular disease: A science advisory from the American Heart Association kidney and cardiovascular disease council; the councils on high blood pressure research, cardiovascular disease in the young, and epidemiology and prevention; and the quality of care and outcomes research interdisciplinary working group: Developed in collaboration with the National Kidney Foundation. Circulation. 2006;114(10):1083–1087. 16894032 10.1161/CIRCULATIONAHA.106.177321

[25] McKhann GM, Knopman DS, Chertkow H, et al The diagnosis of dementia due to Alzheimer’s disease: Recommendations from the National Institute on Aging-Alzheimer’s Association workgroups on diagnostic guidelines for Alzheimer’s disease. Alzheimers Dement. 2011;7(3):263–269. 21514250 10.1016/j.jalz.2011.03.005 PMC3312024

[26] Lin X, Zhang D. Inference in generalized additive mixed models by using smoothing splines. J R Stat Soc Ser B Stat Methodol. 1999;61(2):381–400.

[27] Wood S . Package ‘mgcv.’ Published online 2023. Accessed 11 July 2024. https://cran.r-project.org/web/packages/mgcv/mgcv.pdf

[28] Smithson M, Broomell SB. Compositional data analysis tutorial. Psychol Methods. 2022;29(2):362–378. 35099238 10.1037/met0000464

[29] Rigby RA, Stasinopoulos DM. Generalized additive models for location, scale and shape. J R Stat Soc Ser C Appl Stat. 2005;54(3):507–554.

[30] Ducic I, Yoon J, Buncke G. Chronic postoperative complications and donor site morbidity after sural nerve autograft harvest or biopsy. Microsurgery. 2020;40(6):710–716. 32277511 10.1002/micr.30588 PMC7540447

[31] Friede RL, Beuche W. Combined scatter diagrams of sheath thickness and fibre calibre in human sural nerves: Changes with age and neuropathy. J Neurol Neurosurg Psychiatry. 1985;48(8):749–756. 4031926 10.1136/jnnp.48.8.749 PMC1028445

[32] Parhad IM, Scott JN, Cellars LA, Bains JS, Krekoski CA, Clark AW. Axonal atrophy in aging is associated with a decline in neurofilament gene expression. J Neurosci Res. 1995;41(3):355–366. 7563228 10.1002/jnr.490410308

[33] Hoffman PN, Cleveland DW, Griffin JW, Landes PW, Cowan NJ, Price DL. Neurofilament gene expression: A major determinant of axonal caliber. Proc Natl Acad Sci U S A. 1987;84(10):3472–3476. 3472217 10.1073/pnas.84.10.3472 PMC304893

[34] Shekari A, Fahnestock M. Retrograde axonal transport of BDNF and proNGF diminishes with age in basal forebrain cholinergic neurons. Neurobiol Aging. 2019;84:131–140. 31574357 10.1016/j.neurobiolaging.2019.07.018

[35] Kihara M, Nakasaka Y, Mitsui Y, Takahashi M, Schmelzer JD. Aging differentially modifies sensitivity of nerve blood flow to vasocontractile agents (endothelin-1, noradrenaline and angiotensin II) in sciatic nerve. Mech Ageing Dev. 2000;114(1):5–14. 10731577 10.1016/s0047-6374(99)00115-3

[36] Cruz-Jentoft AJ, Sayer AA. Sarcopenia. The Lancet. 2019;393(10191):2636–2646. 10.1016/S0140-6736(19)31138-9 31171417

[37] García-Piqueras J, García-Mesa Y, Cárcaba L, et al Ageing of the somatosensory system at the periphery: Age-related changes in cutaneous mechanoreceptors. J Anat. 2019;234(6):839–852. 30924930 10.1111/joa.12983 PMC6539748

[38] Hastings RL, Avila MF, Suneby E, et al Cellular and molecular evidence that synaptic Schwann cells contribute to aging of mouse neuromuscular junctions. Aging Cell. 2023;22(11):e13981. 37771191 10.1111/acel.13981 PMC10652323

[39] Rodrigues ACZ, Messi ML, Wang ZM, et al The sympathetic nervous system regulates skeletal muscle motor innervation and acetylcholine receptor stability. Acta Physiol Oxf Engl. 2019;225(3):e13195. 10.1111/apha.13195 PMC7224611 30269419

[40] Rodrigues ACZ, Messi ML, Wang ZM, Bonilla HJ, Freeman WM, Delbono O. Long-term, induced expression of Hand2 in peripheral sympathetic neurons ameliorates sarcopenia in geriatric mice. J Cachexia Sarcopenia Muscle. 2021;12(6):1908–1924. 34546662 10.1002/jcsm.12790 PMC8718059

[41] Rodrigues ACZ, Wang ZM, Messi ML, et al Heart and neural crest derivative 2-induced preservation of sympathetic neurons attenuates sarcopenia with aging. J Cachexia Sarcopenia Muscle. 2021;12(1):91–108. 33258279 10.1002/jcsm.12644 PMC7890150

[42] Delbono O, Wang ZM, Messi ML. Brainstem noradrenergic neurons: Identifying a hub at the intersection of cognition, motility, and skeletal muscle regulation. Acta Physiol Oxf Engl. 2022;236(3):e13887. 10.1111/apha.13887 PMC9588743 36073023

[43] Oveisgharan S, Wang T, Barnes LL, Schneider JA, Bennett DA, Buchman AS. The time course of motor and cognitive decline in older adults and their associations with brain pathologies: A multicohort study. Lancet Healthy Longev. 2024;5(5):e336–e345. 38582095 10.1016/S2666-7568(24)00033-3 PMC11129202

[44] Buchman AS, Yu L, Klein HU, et al Proteome-wide discovery of cortical proteins that may provide motor resilience to offset the negative effects of pathologies in older adults. J Gerontol A Biol Sci Med Sci. 2023;78(3):494–503. 35512265 10.1093/gerona/glac105 PMC9977240

[45] Yu L, Boyle P, Wilson RS, et al A random change point model for cognitive decline in Alzheimer’s disease and mild cognitive impairment. Neuroepidemiology. 2012;39(2):73–83. 22814083 10.1159/000339365 PMC3484884

[46] Bennett DA, Wilson RS, Schneider JA, et al Education modifies the relation of AD pathology to level of cognitive function in older persons. Neurology. 2003;60(12):1909–1915. 12821732 10.1212/01.wnl.0000069923.64550.9f

[47] Oveisgharan S, Wilson RS, Yu L, Schneider JA, Bennett DA. Association of early-life cognitive enrichment with Alzheimer disease pathological changes and cognitive decline. JAMA Neurol. 2020;77(10):1217–1224. 32597941 10.1001/jamaneurol.2020.1941 PMC7325069

[48] Schmid ET, Pyo JH, Walker DW. Neuronal induction of BNIP3-mediated mitophagy slows systemic aging in Drosophila. Nat Aging. 2022;2(6):494–507. 36213625 10.1038/s43587-022-00214-y PMC9540997

[49] Grover-Johnson N, Spencer PS. Peripheral nerve abnormalities in aging rats. J Neuropathol Exp Neurol. 1981;40(2):155–165. 7463100 10.1097/00005072-198103000-00007

[50] Beirowski B, Babetto E, Golden JP, et al Metabolic regulator LKB1 is crucial for Schwann cell-mediated axon maintenance. Nat Neurosci. 2014;17(10):1351–1361. 25195104 10.1038/nn.3809 PMC4494117

[51] Baraban M, Mensch S, Lyons DA. Adaptive myelination from fish to man. Brain Res. 2016;1641(Pt A):149–161. 26498877 10.1016/j.brainres.2015.10.026 PMC4907128

[52] Pease-Raissi SE, Chan JR. Building a (w)rapport between neurons and oligodendroglia: Reciprocal interactions underlying adaptive myelination. Neuron. 2021;109(8):1258–1273. 33621477 10.1016/j.neuron.2021.02.003 PMC8068592

[53] Fahrenkamp I, Friede RL. Characteristic variations of relative myelin sheath thickness in 11 nerves of the rat. Anat Embryol (Berl). 1987;177(2):115–121. 3434842 10.1007/BF00572535

[54] Moriyama H, Amano K, Itoh M, Shimada K, Otsuka N. Morphometric aspects of peripheral nerves in adults and the elderly. J Peripher Nerv Syst JPNS. 2007;12(3):205–209. 17868247 10.1111/j.1529-8027.2007.00140.x

[55] Tani H, Goto N, Goto J, Lu S, Ma X. Morphometric analysis of the human tibial nerve and the ageing process. Okajimas Folia Anat Jpn. 2001;78(2–3):61–64. 11732206 10.2535/ofaj1936.78.2-3_61

[56] Thomas PK, King RH, Sharma AK. Changes with age in the peripheral nerves of the rat. An ultrastructural study. Acta Neuropathol (Berl). 1980;52(1):1–6. 7435152 10.1007/BF00687222

