# Supplementary material for: Morphometric characteristics of tibial nerve and their relationship with age
Source: Brain Commun. 2025 Jul 7;7(4):fcaf267. doi: 10.1093/braincomms/fcaf267 (PMC12272163; doi:10.1093/braincomms/fcaf267)
Supplement: fcaf267_Supplementary_Data [file fcaf267_supplementary_data.docx]

| **Content** | **Pages** |
| --- | --- |
| **Supplementary Figure 1. Cross section of tibial nerve in an 84- (A) and 95- (B) year-old participant.** | **4** |
| **Supplementary Table 1. Association of age at death with person-specific averages of the morphometric characteristics of tibial nerve.** | **5** |
| **Supplementary Table 2. Averages of percentages of myelinated nerve fibers in the 6 diameter groups.** | **6** |
| **Supplementary Table 3. The dispersion submodel of the compositional analysis of the association of age at death with the percentage of myelinated nerve fibers in the 6 diameter groups.** | **7** |
| **Supplementary Table 4. The compositional analysis of the association of age at death with the percentage of myelinated nerve fibers in the 6 diameter groups with different terms in the location and dispersion submodels.** | **8** |
| **Supplementary Figure 2. Association of age at death with density of myelinated nerve fibers.** | **9** |
| **Supplementary Table 5. Descriptive statistics of the myelinated nerve fibers after removing 37729 myelinated nerve fibers with g-ratio=1.** | **10** |
| **Supplementary Figure 3. Distribution of morphometric characteristics of myelinated nerve fibers (n=716518) after removing 37729 myelinated nerve fibers with g-ratio=1.** | **11** |
| **Supplementary Figure 4. Associations of axon diameter with myelin thickness after removing 37729 myelinated nerve fibers with g-ratio=1.** | **12** |
| **Supplementary Table 6. Associations of age at death with person-specific averages of the morphometric characteristics of the tibial nerve, calculated after removing 37729 myelinated nerve fibers with g-ratio=1.** | **13** |
| **Supplementary Figure 5. Scatter plots of age at death in relation to person-specific averages of myelinated nerve fiber characteristics, calculated after removing 37729 myelinated nerve fibers with g-ratio=1.** | **14** |
| **Supplementary Table 7. Association of age at death with the percentage of myelinated nerve fibers of different diameters, calculated after removing 37729 myelinated nerve fibers with g-ratio=1.** | **15** |
| **Supplementary Figure 6. Association of age at death with density of myelinated nerve fibers, calculated after removing 37729 myelinated nerve fibers with g-ratio=1.** | **16** |
| **Supplementary Table 8. Association of age at death with g-ratio of the myelinated nerve fibers classified by their diameter after removing 37729 myelinated nerve fibers with g-ratio=1.** | **17** |
| **Supplementary Table 9. Descriptive statistics of the myelinated nerve fibers after removing 32707 myelinated nerve fibers with a diameter < 1 µm.** | **18** |
| **Supplementary Figure 7. Distribution of morphometric characteristics of myelinated nerve fibers (n=721540) after removing 32707 myelinated nerve fibers with a diameter < 1 µm.** | **19** |
| **Supplementary Figure 8. Associations of axon diameter with myelin thickness after removing 32707 myelinated nerve fibers with a diameter < 1 µm.** | **20** |
| **Supplementary Table 10. Associations of age at death with person-specific averages of the morphometric characteristics of the tibial nerve, calculated after removing 32707 myelinated nerve fibers with a diameter < 1 µm.** | **21** |
| **Supplementary Figure 9. Scatter plots of age at death in relation to person-specific averages of myelinated nerve fiber characteristics, calculated after removing 32707 myelinated nerve fibers with a diameter < 1 µm.** | **22** |
| **Supplementary Table 11. Association of age at death with the percentage of myelinated nerve fibers of different diameters, calculated after removing 32707 myelinated nerve fibers with a diameter < 1 µm.** | **23** |
| **Supplementary Figure 10. Association of age at death with density of myelinated nerve fibers, calculated after removing 32707 myelinated nerve fibers with a diameter < 1 µm.** | **24** |
| **Supplementary Table 12. Association of age at death with g-ratio of the myelinated nerve fibers classified by their diameter after removing 32707 myelinated nerve fibers with a diameter < 1 µm.** | **25** |
| **Supplementary Table 13. Descriptive statistics of the myelinated nerve fibers after removing 66550 myelinated nerve fibers with an axon diameter < 0.8 µm.** | **26** |
| **Supplementary Figure 11. Distribution of morphometric characteristics of myelinated nerve fibers (n=687697) after removing 66550 myelinated nerve fibers with an axon diameter < 0.8 µm.** | **27** |
| **Supplementary Figure 12. Associations of axon diameter with myelin thickness after removing 66550 myelinated nerve fibers with an axon diameter < 0.8 µm.** | **28** |
| **Supplementary Table 14. Associations of age at death with person-specific averages of the morphometric characteristics of the tibial nerve, calculated after removing 66550 myelinated nerve fibers with an axon diameter < 0.8 µm.** | **29** |
| **Supplementary Figure 13. Scatter plots of age at death in relation to person-specific averages of myelinated nerve fiber characteristics, calculated after removing 66550 myelinated nerve fibers with an axon diameter < 0.8 µm.** | **30** |
| **Supplementary Table 15. Association of age at death with the percentage of myelinated nerve fibers of different diameters, calculated after removing 66550 myelinated nerve fibers with an axon diameter < 0.8 µm.** | **31** |
| **Supplementary Figure 14. Association of age at death with density of myelinated nerve fibers, calculated after removing 66550 myelinated nerve fibers with an axon diameter < 0.8 µm.** | **32** |
| **Supplementary Table 16. Association of age at death with g-ratio of the myelinated nerve fibers classified by their diameter after removing 66550 myelinated nerve fibers with an axon diameter < 0.8 µm.** | **33** |
| **Supplementary references** | **34** |

**Supplementary Figure 1. Cross section of tibial nerve in an 84- (A) and 95- (B) year-old participant.**


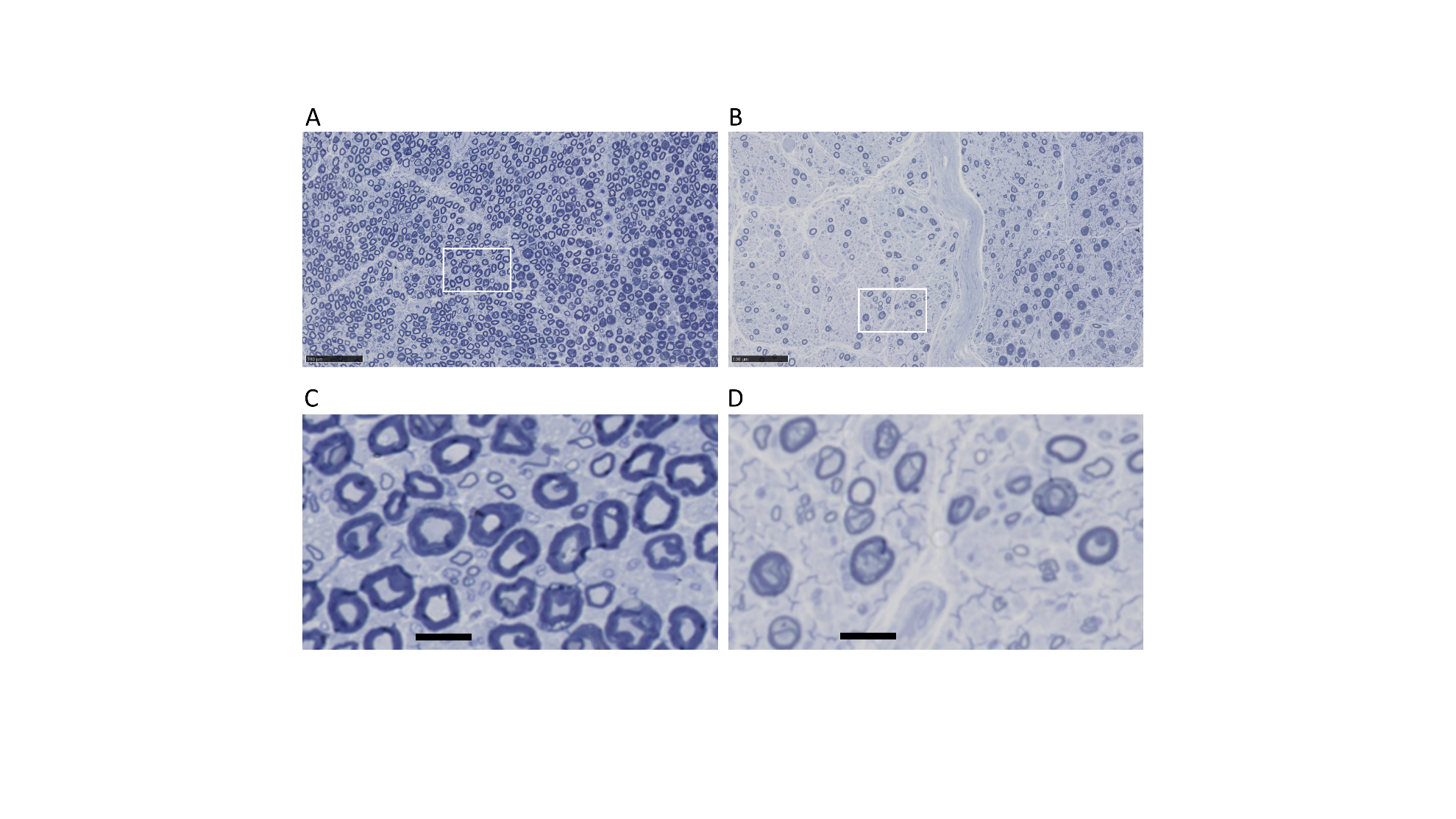


The images A-B were taken using a 40X microscope magnification and a 20X digital zoom, and a scale bar indicating 100 µm is included. The images C-D are the closeup of the rectangular areas in the images A-B, respectively, with an included scale bar indicating 15 µm. Each closed curve represents a myelinated nerve fiber with a peripheral dark blue ribbon, indicative of myelin, and a central pale segment indicative of axon. The myelinated nerve fibers are larger in the younger (**A**) compared to an older (**B**) participant.

**Supplementary Table 1. Association of age at death with person-specific averages of the morphometric characteristics of tibial nerve.**

| **Model term** | **Estimate (SE), p-value** | | | |
| --- | --- | --- | --- | --- |
|  | **Myelinated nerve fiber diameter** | **Axon diameter** | **Myelin thickness** | **g-ratio** |
| Intercept | 4.927 (0.088), <0.001 | 2.080 (0.037), <0.001 | 1.424 (0.028), <0.001 | 0.458 (0.004), <0.001 |
| Age at death | -0.041 (0.014), 0.004 | -0.014 (0.006), 0.017 | -0.013 (0.004), 0.003 | 0.001 (0.001), 0.185 |
| Sex (women vs. men) | 0.072 (0.168), 0.667 | 0.079 (0.071), 0.263 | -0.003 (0.052), 0.948 | 0.009 (0.007), 0.188 |

The second to fifth columns’ numbers are derived from 4 separate linear regression models with the person-specific averages of the morphometric characteristics of tibial nerve as the outcome and the model terms listed in the left column.

**Supplementary Table 2. Averages of percentages of myelinated nerve fibers in the 6 diameter groups.**

| **Category of Myelinated nerve fiber diameter** | **Percentage** | |
| --- | --- | --- |
|  | **Mean (SD)** | **Range** |
| <2 µm | 11.7% (6.3) | 3.9% – 48.4% |
| 2 to <4 µm | 40.7% (9.9) | 13.9% – 65.1% |
| 4 to <6 µm | 21.2% (5.6) | 3.7% – 38.5% |
| 6 to <8 µm | 8.5% (3.2) | 1.7% – 18.1% |
| 8 to <10 µm | 8.1% (4.8) | 0.9% – 27.1% |
| ≥10 µm | 9.9% (6.8) | 0.6% – 44.6% |

To calculate the percentages, each myelinated nerve fiber was classified into six groups based on their diameter. Then, for each person and each category, the percentage of myelinated nerve fibers in the size group was calculated. For example, 7075 myelinated nerve fibers were studied in a participant, and 595 of them had a diameter < 2 µm. The percentage of myelinated nerve fibers <2 µm in the participant was (595/7075)×100 = 8.4%. Summaries of these percentages across the 140 persons are shown.

**Supplementary Table 3. The dispersion submodel of the compositional analysis of the association of age at death with the percentage of myelinated nerve fibers in the 6 diameter groups.**

| **Model terms** | **Estimate (SE), p-value** |
| --- | --- |
| Intercept | -0.784 (.062), <0.001 |
| Age at death | 0.004 (0.010), 0.702 |
| Sex | 0.042 (0.062), 0.495 |
| Percent of myelinated nerve fibers of 2 to <4µm / Percent of myelinated nerve fibers of <2µm | REF. |
| Percent of myelinated nerve fibers of 4 to <6µm / Percent of myelinated nerve fibers of <2µm | 0.392 (0.085), <0.001 |
| Percent of myelinated nerve fibers of 6 to <8µm / Percent of myelinated nerve fibers of <2µm | 0.375 (0.085), <0.001 |
| Percent of myelinated nerve fibers of 8 to <10µm / Percent of myelinated nerve fibers of <2µm | 0.569 (0.085), <0.001 |
| Percent of myelinated nerve fibers of ≥10µm / Percent of myelinated nerve fibers of <2µm | 0.780 (0.085), <0.001 |
| Age×( Percent of myelinated nerve fibers of 4 to <6µm / Percent of myelinated nerve fibers of <2µm) | 0.018 (0.013), 0.176 |
| Age×( Percent of myelinated nerve fibers of 6 to <8µm / Percent of myelinated nerve fibers of <2µm) | 0.019 (0.013), 0.160 |
| Age×( Percent of myelinated nerve fibers of 8 to <10µm / Percent of myelinated nerve fibers of <2µm) | 0.022 (0.014), 0.109 |
| Age×( Percent of myelinated nerve fibers of ≥10µm / Percent of myelinated nerve fibers of <2µm) | 0.020 (0.014), 0.168 |

The second column numbers are derived from a heteroscedastic normal distribution log-ratio regression model with the model terms listed in the left column and the outcome is ratio of the percentage of myelinated nerve fibers in 2-4, 4-6, 6-8, 8-10, or ≥10µm to percentage of myelinated nerve fibers with the size of 0-2µm.

**Supplementary Table 4. The compositional analysis of the association of age at death with the percentage of myelinated nerve fibers in the 6 diameter groups with different terms in the location and dispersion submodels.**

| **Model terms** | **Location submodel** | **Dispersion submodel** |
| --- | --- | --- |
|  | **Estimate (SE), p-value** | |
| Intercept | 1.311 (.042), <0.001 | -0.775 (.063), <0.001 |
| Age at death | 0.001 (0.007), 0.823 | 0.019 (0.004), <0.001 |
| Sex | 0.025 (0.056), 0.657 | 0.042 (0.062), 0.498 |
| Percent of myelinated nerve fibers of 2 to <4µm / Percent of myelinated nerve fibers of <2µm | REF. | REF. |
| Percent of myelinated nerve fibers of 4 to <6µm / Percent of myelinated nerve fibers of <2µm | -0.660 (0.071), <0.001 | 0.384 (0.085), <0.001 |
| Percent of myelinated nerve fibers of 6 to <8µm / Percent of myelinated nerve fibers of <2µm | -1.605 (0.070), <0.001 | 0.367 (0.085), <0.001 |
| Percent of myelinated nerve fibers of 8 to <10µm / Percent of myelinated nerve fibers of <2µm | -1.758 (0.080), <0.001 | 0.562 (0.085), <0.001 |
| Percent of myelinated nerve fibers of ≥10µm /  Percent of myelinated nerve fibers of <2µm | -1.620 (0.095), <0.001 | 0.772 (0.085), <0.001 |
| Age×(Percent of myelinated nerve fibers of 4 to <6µm / Percent of myelinated nerve fibers of <2µm) | -0.007 (0.012), 0.547 | NA |
| Age×(Percent of myelinated nerve fibers of 6 to <8µm / Percent of myelinated nerve fibers of <2µm) | -0.018 (0.012), 0.123 | NA |
| Age×(Percent of myelinated nerve fibers of 8 to <10µm / Percent of myelinated nerve fibers of <2µm) | -0.035 (0.013), 0.009 | NA |
| Age×(Percent of myelinated nerve fibers of ≥10µm / Percent of myelinated nerve fibers of <2µm) | -0.039 (0.016), 0.015 | NA |

Because the interaction terms between age and diameter groups were not significant in the dispersion submodel of Supplementary Table 3, in a heteroscedastic model we removed the interaction terms from the dispersion submodel.

**Supplementary Figure 2. Association of age at death with density of myelinated nerve fibers.**

**
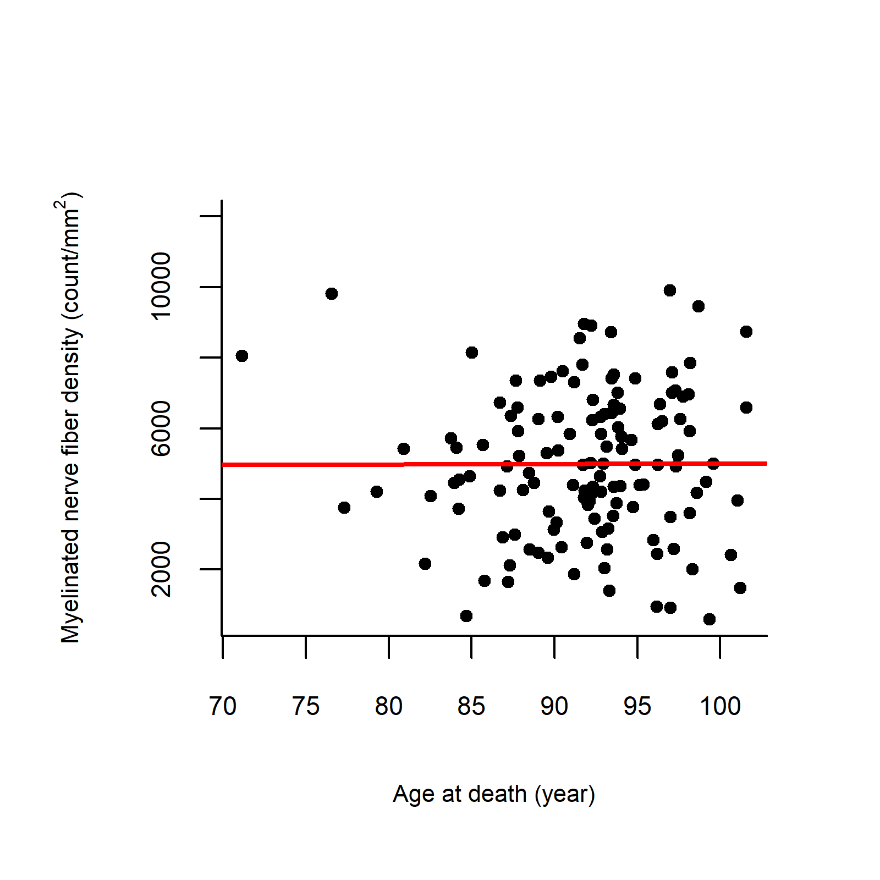
**

In a linear regression model, myelinated nerve fiber density was the outcome and age at death as the model term, controlled for sex. The sample size was 134 because the nerve area was missed in 9 participants. Each dot represents the myelinated nerve fiber density of the tibial nerve and age at death of a participant.

**Supplementary Table 5. Descriptive statistics of the myelinated nerve fibers after removing 37729 myelinated nerve fibers with g-ratio=1.**

| **Characteristics** | **Mean (SD) or count** |
| --- | --- |
| **The myelinated nerve fibers altogether** |  |
| Number of the examined myelinated nerve fibers | 716518 |
| Diameter of myelinated nerve fiber, (μm) | 5.1 (3.0) |
| Axon diameter, (μm) | 2.1 (1.4) |
| Myelin thickness, (μm) | 1.5 (0.9) |
| G-ratio | 0.42 (0.11) |
| **Characteristics at person-level** |  |
| Number of the myelinated nerve fibers per participant | 5118.0 (3334.1) |
| Person-specific average of myelinated nerve fiber diameter, (μm) | 5.2 (0.9) |
| Person-specific average of axon diameter, (μm) | 2.1 (0.4) |
| Person-specific average of myelin thickness, (μm) | 1.5 (0.3) |
| Person-specific average of g-ratio | 0.42 (0.02) |
| Myelinated nerve fiber density, (count/mm^2^) | 4721.3 (1970.4) |

**Supplementary Figure 3. Distribution of morphometric characteristics of myelinated nerve fibers (n=716518) after removing 37729 myelinated nerve fibers with g-ratio=1.**


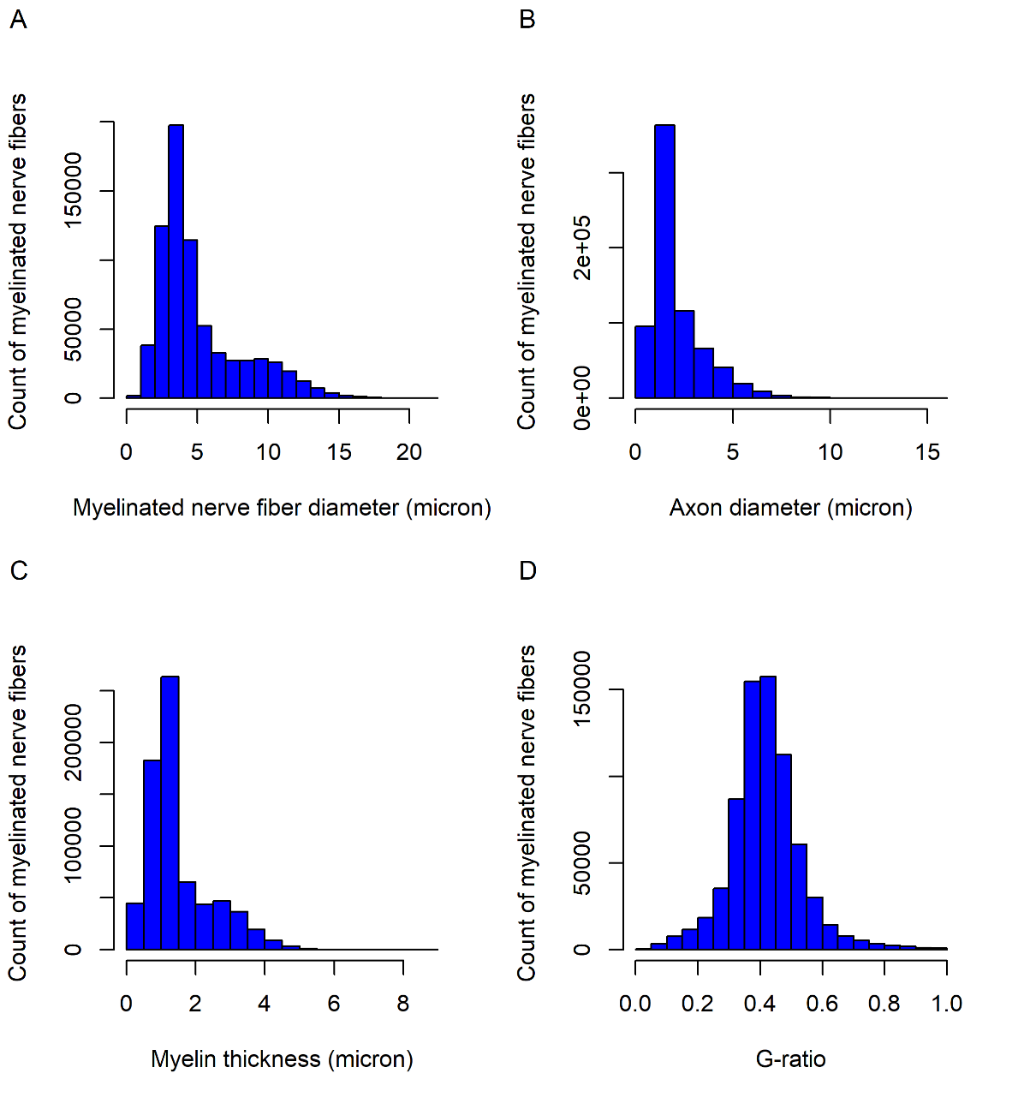


Histogram of 716518 myelinated nerve fibers illustrating their distribution by the diameter of the myelinated nerve fiber (A), axonal diameter (B), myelin thickness (C), and g-ratio (D). The 716518 myelinated nerve fibers from selected from the pool of 754247 nerve fibers after excluding myelinated nerve fibers with g-ratio=1.

**Supplementary Figure 4. Associations of axon diameter with myelin thickness after removing 37729 myelinated nerve fibers with g-ratio=1.**

**
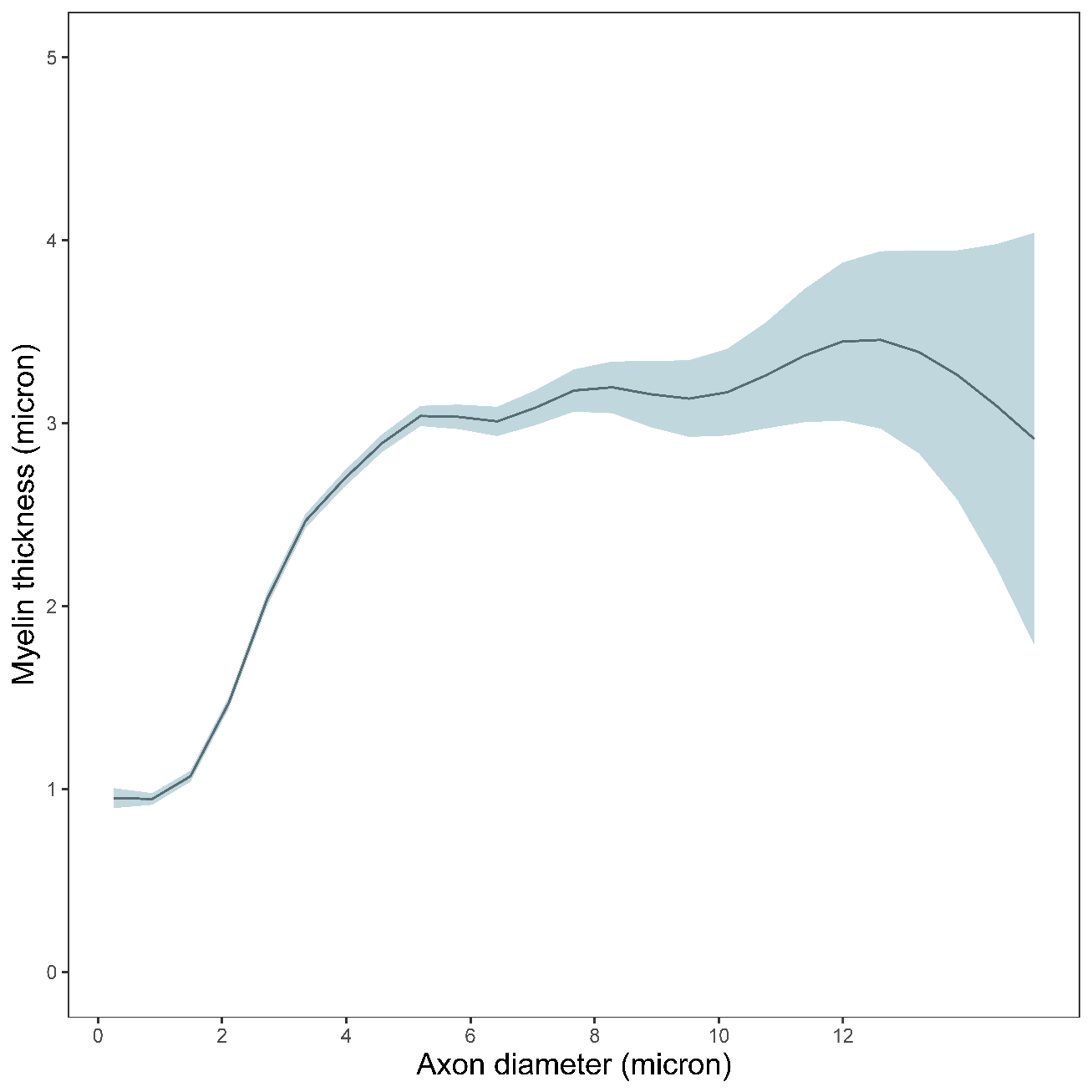
**

Association between axon diameter and myelin thickness derived from a generalized additive mixed model, using the *gamm* function from *mgcv* package^1^ in R, analyzing 14000 myelinated nerve fibers, which included 100 randomly selected myelinated nerve fibers from each of 140 participants. The 14000 myelinated nerve fibers were selected from 716518 myelinated nerve fibers, which were prepared by excluding 37729 out of the pool of 754247 myelinated nerve fibers that had a g-ratio=1. The Y-axis is the estimated myelin thickness. The shaded blue area around the curve indicates 95% confidence interval of myelin thickness.

**Supplementary Table 6. Associations of age at death with person-specific averages of the morphometric characteristics of the tibial nerve, calculated after removing 37729 myelinated nerve fibers with g-ratio=1.**

| **Morphometric characteristics** | **Associations of age at death** | |
| --- | --- | --- |
|  | **Spearman correlation coefficient, p-value** | **Estimate (SE), p-value** |
| **Person-specific average of myelinated nerve fiber diameter** | -0.23, 0.007 | -0.042 (0.014), 0.004 |
| **Person-specific average of axon diameter** | -0.16, 0.056 | -0.015 (0.006), 0.017 |
| **Person-specific average of myelin thickness** | -0.26, 0.002 | -0.014 (0.004), 0.002 |
| **Person-specific average of g-ratio** | 0.10, 0.23 | 0.0005 (0.0004), 0.18 |
| **Myelinated nerve fiber density** | 0.08, 0.34 | 6.77 (32.48), 0.84 |

In 5 separate linear regression models, each of the person-specific averages of the morphometric characteristics of the tibial nerve was the outcome, and age at death and sex were the model terms. The person-specific averages of the morphometric characteristics were calculated after exclusion of 37729 myelinated nerve fibers that had g-ratio=1. Age at death was associated with smaller myelinated nerve fibers, smaller axons, and thinner myelin, but g-ratio and myelinated nerve fiber density were not related to age at death.

**Supplementary Figure 5. Scatter plots of age at death in relation to person-specific averages of myelinated nerve fiber characteristics, calculated after removing 37729 myelinated nerve fibers with g-ratio=1.**

**
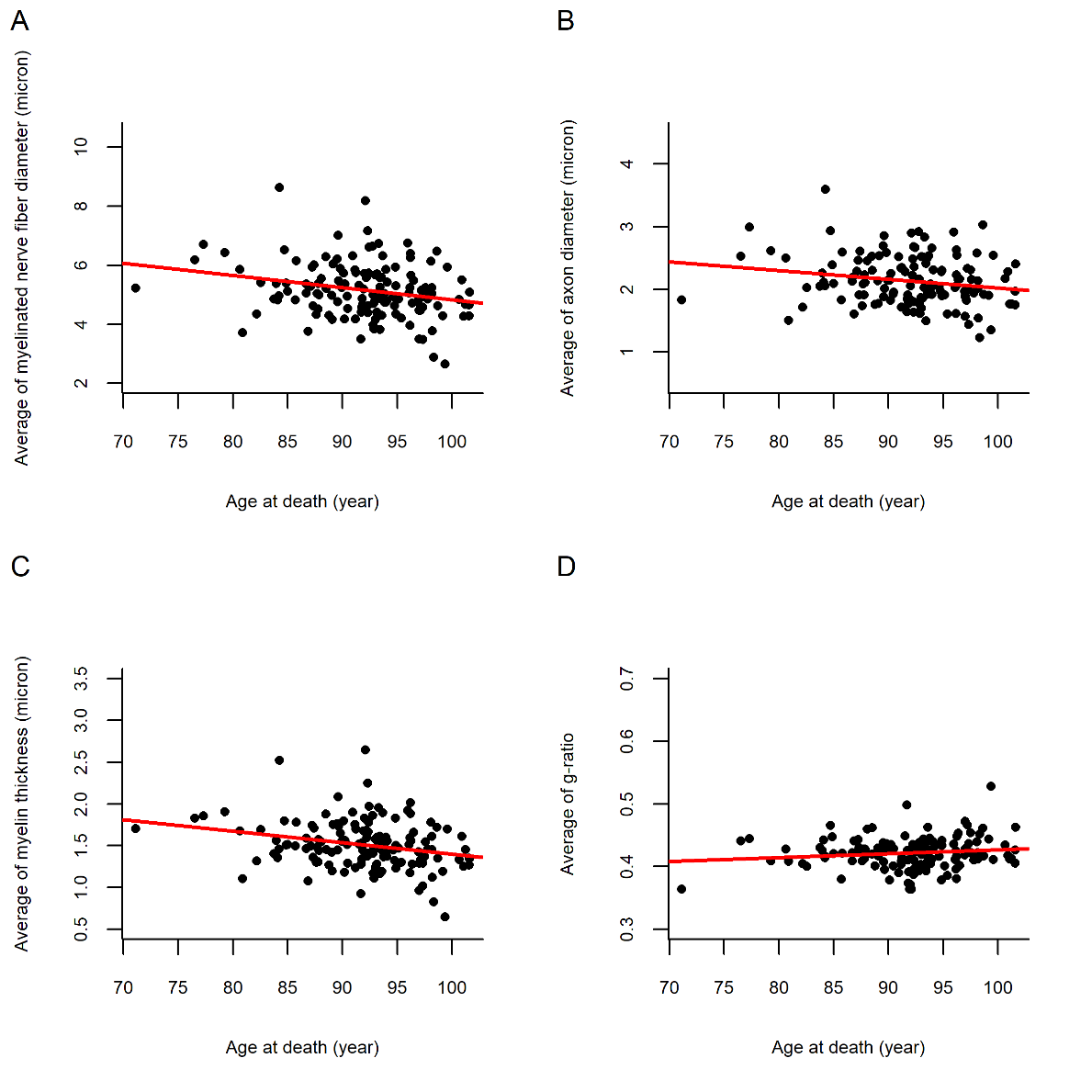
**

The 4 scatter plots illustrate the association between age at death and person-specific averages of myelinated nerve fibers diameter (A), axonal diameter (B), myelin thickness (C), and g-ratio (D). The person-specific averages were calculated from 716518 myelinated nerve fibers, which were prepared by excluding 37729 out of the pool of 754247 myelinated nerve fibers that had a g-ratio=1. Each dot represents a participant’s age at death (x-axis) and one of the person-specific averages of the morphometric characteristics. The red lines are derived from linear regression models including 140 participants.

**Supplementary Table 7. Association of age at death with the percentage of myelinated nerve fibers of different diameters, calculated after removing 37729 myelinated nerve fibers with g-ratio=1.**

| **Model terms** | **Estimate (SE), p-value** |
| --- | --- |
| Intercept | 1.996 (0.041), <0.001 |
| Age at death | -0.001 (0.007), 0.892 |
| Sex | 0.051 (0.058), 0.375 |
| Percent of myelinated nerve fibers 2 to <4µm / Percent of myelinated nerve fibers <2µm | REF. |
| Percent of myelinated nerve fibers 4 to <6µm / Percent of myelinated nerve fibers <2µm | -0.656 (0.072), <0.001 |
| Percent of myelinated nerve fibers 6 to <8µm / Percent of myelinated nerve fibers <2µm | -1.600 (0.075), <0.001 |
| Percent of myelinated nerve fibers 8 to <10µm / Percent of myelinated nerve fibers <2µm | -1.753 (0.085), <0.001 |
| Percent of myelinated nerve fibers ≥10µm / Percent of myelinated nerve fibers <2µm | -1.616 (0.099), <0.001 |
| Age × (Percent of myelinated nerve fibers 4 to <6µm / Percent of myelinated nerve fibers <2µm) | -0.008 (0.012), 0.544 |
| Age × (Percent of myelinated nerve fibers 6 to <8µm / Percent of myelinated nerve fibers <2µm) | -0.018 (0.013), 0.160 |
| Age × (Percent of myelinated nerve fibers 8 to <10µm / Percent of myelinated nerve fibers <2µm) | -0.035 (0.015), 0.017 |
| Age × (Percent of myelinated nerve fibers ≥10µm / Percent of myelinated nerve fibers <2µm) | -0.040 (0.017), 0.019 |

**Supplementary Figure 6. Association of age at death with density of myelinated nerve fibers, calculated after removing 37729 myelinated nerve fibers with g-ratio=1.**

**
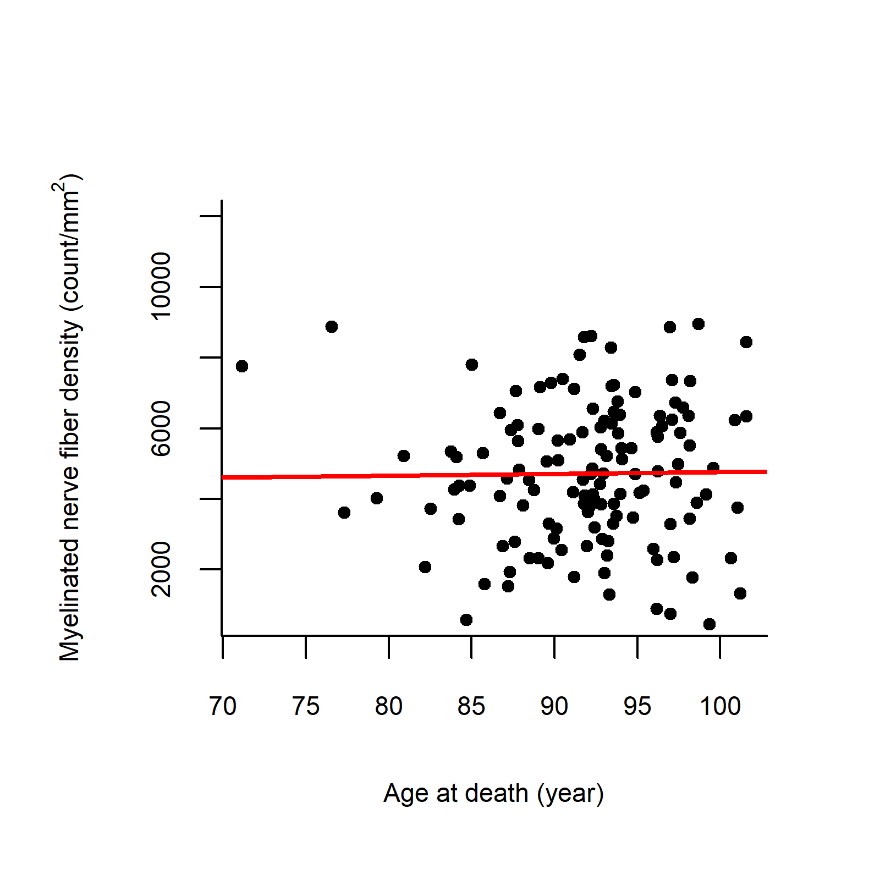
**

In a linear regression model, myelinated nerve fiber density was the outcome and age at death as the model term, controlled for sex. Myelinated nerve fiber density was calculated after excluding myelinated nerve fibers with g-ratio = 1. The sample size was 134 because the nerve area was missed in 9 participants. Each dot represents the myelinated nerve fiber density of the tibial nerve and age at death of a participant.

**Supplementary Table 8. Association of age at death with g-ratio of the myelinated nerve fibers classified by their diameter after removing 37729 myelinated nerve fibers with g-ratio=1.**

| **Model terms** | **Estimate (SE), p-value** |
| --- | --- |
| Intercept | 0.425 (0.003), <0.001 |
| Age at death | 0.0004 (0.0004), 0.329 |
| Sex | -0.010 (0.002), <0.001 |
| Myelinated nerve fibers <2µm | 0.153 (0.007), <0.001 |
| Myelinated nerve fibers 2 to <4 µm | -0.012 (0.003), <0.001 |
| Myelinated nerve fibers 4 to <6 µm | -0.006 (0.003), 0.076 |
| Myelinated nerve fibers 6 to <8µm | -0.007 (0.004), 0.079 |
| Myelinated nerve fibers 8 to <10µm | -0.028 (0.004), <0.001 |
| Myelinated nerve fibers ≥10µm | **REF** |
| Age×Myelinated nerve fibers <2µm | -0.0009 (0.0012), 0.468 |
| Age×Myelinated nerve fibers 2 to <4µm | -0.0009 (0.0005), 0.084 |
| Age×Myelinated nerve fibers 4 to <6µm | -0.0002 (0.0006), 0.761 |
| Age×Myelinated nerve fibers 6 to <8µm | 0.0006 (0.0007), 0.407 |
| Age×Myelinated nerve fibers 8 to <10µm | 0.0004 (0.0006), 0.559 |

**Supplementary Table 9. Descriptive statistics of the myelinated nerve fibers after removing 32707 myelinated nerve fibers with a diameter < 1 µm.**

| **Characteristics** | **Mean (SD) or count** |
| --- | --- |
| **The myelinated nerve fibers altogether** |  |
| Number of the examined myelinated nerve fibers | 721540 |
| Diameter of myelinated nerve fiber, (μm) | 5.1 (3.0) |
| Axon diameter, (μm) | 2.1 (1.4) |
| Myelin thickness, (μm) | 1.5 (0.9) |
| G-ratio | 0.42 (0.13) |
| **Characteristics at person-level** |  |
| Number of the myelinated nerve fibers per participant | 5153.9 (3343.8) |
| Person-specific average of myelinated nerve fiber diameter, (μm) | 5.1 (0.9) |
| Person-specific average of axon diameter, (μm) | 2.1 (0.4) |
| Person-specific average of myelin thickness, (μm) | 1.5 (0.3) |
| Person-specific average of g-ratio | 0.43 (0.03) |
| Myelinated nerve fiber density, (count/mm^2^) | 4760.1 (1978.6) |

**Supplementary Figure 7. Distribution of morphometric characteristics of myelinated nerve fibers (n=721540) after removing 32707 myelinated nerve fibers with a diameter < 1 µm.**

**
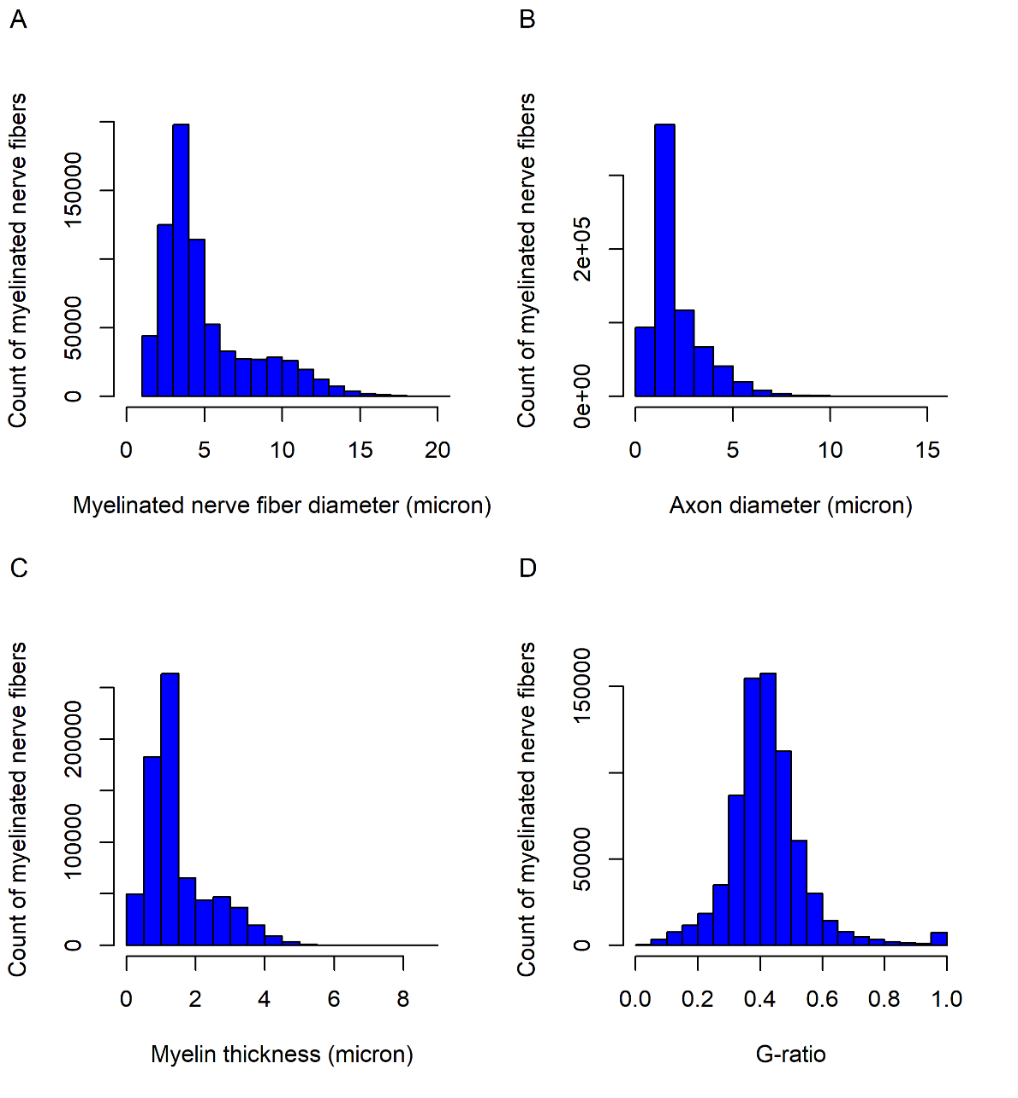
**

Histogram of 721540 myelinated nerve fibers illustrating their distribution by the diameter of the myelinated nerve fiber (A), axonal diameter (B), myelin thickness (C), and g-ratio (D). The 721540 myelinated nerve fibers from selected from the pool of 754247 nerve fibers after excluding myelinated nerve fibers with a diameter < 1 µm.

**Supplementary Figure 8. Associations of axon diameter with myelin thickness after removing 32707 myelinated nerve fibers with a diameter < 1 µm.**


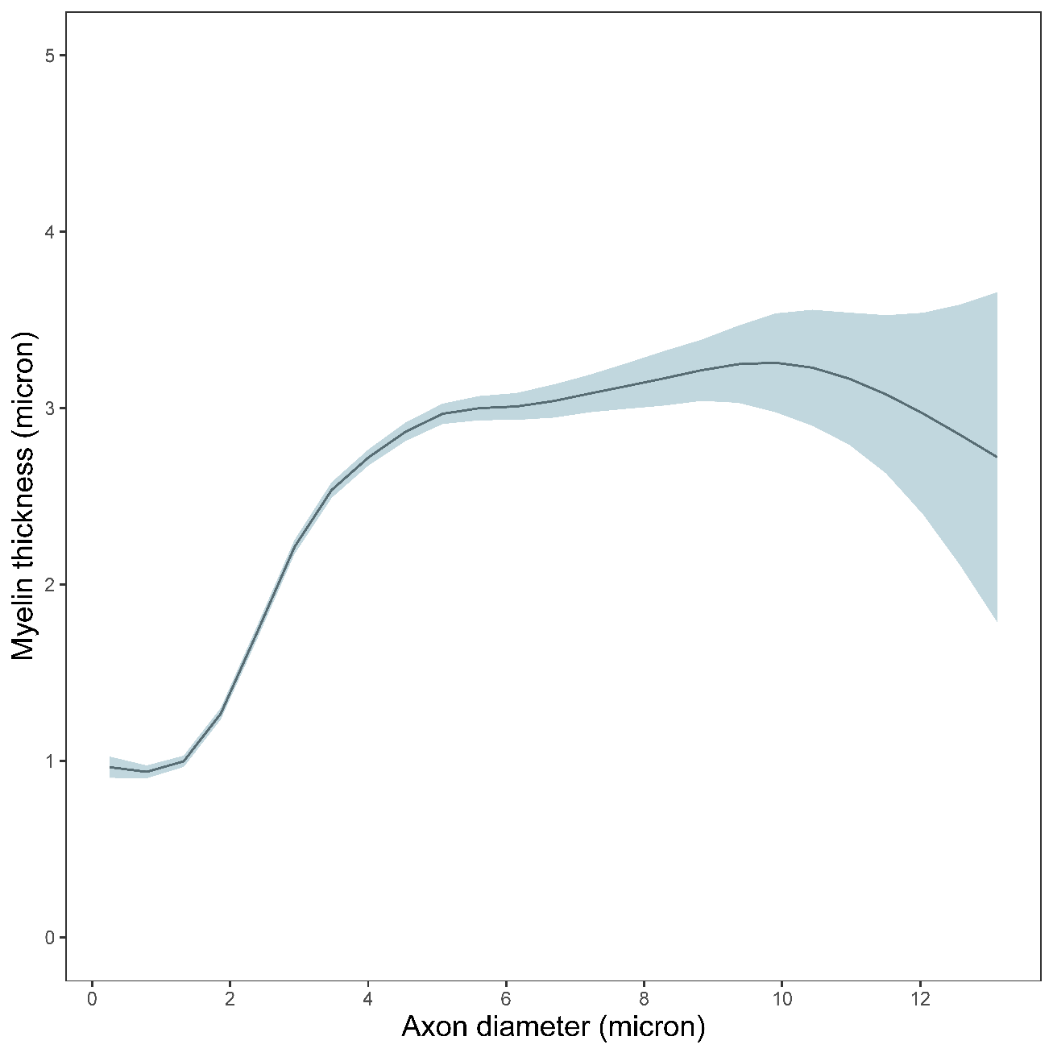


Association between axon diameter and myelin thickness derived from a generalized additive mixed model, using the *gamm* function from *mgcv* package^1^ in R, analyzing 14000 myelinated nerve fibers, which included 100 randomly selected myelinated nerve fibers from each of 140 participants. The 14000 myelinated nerve fibers were selected from 721540 myelinated nerve fibers, which were prepared by excluding 32707 out of the pool of 754247 myelinated nerve fibers that had a diameter < 1 µm. The Y-axis is the estimated myelin thickness. The shaded blue area around the curve indicates 95% confidence interval of myelin thickness.

**Supplementary Table 10. Associations of age at death with person-specific averages of the morphometric characteristics of the tibial nerve, calculated after removing 32707 myelinated nerve fibers with a diameter < 1 µm.**

| **Morphometric characteristics** | **Associations of age at death** | |
| --- | --- | --- |
|  | **Spearman correlation coefficient, p-value** | **Estimate (SE), p-value** |
| **Person-specific average of myelinated nerve fiber diameter** | -0.23, 0.006 | -0.041 (0.014), 0.004 |
| **Person-specific average of axon diameter** | -0.16, 0.062 | -0.014 (0.006), 0.017 |
| **Person-specific average of myelin thickness** | -0.26, 0.002 | -0.014 (0.004), 0.002 |
| **Person-specific average of g-ratio** | 0.09, 0.30 | 0.0005 (0.0004), 0.22 |
| **Myelinated nerve fiber density** | 0.08, 0.35 | 6.64 (32.61), 0.84 |

In 5 separate linear regression models, each of the person-specific averages of the morphometric characteristics of the tibial nerve was the outcome, and age at death and sex were the model terms. The person-specific averages of the morphometric characteristics were calculated after exclusion of 32707 myelinated nerve fibers with a diameter < 1 µm. Age at death was associated with smaller myelinated nerve fibers, smaller axons, and thinner myelin, but g-ratio and myelinated nerve fiber density were not related to age at death.

**Supplementary Figure 9. Scatter plots of age at death in relation to person-specific averages of myelinated nerve fiber characteristics, calculated after removing 32707 myelinated nerve fibers with a diameter < 1 µm.**

**
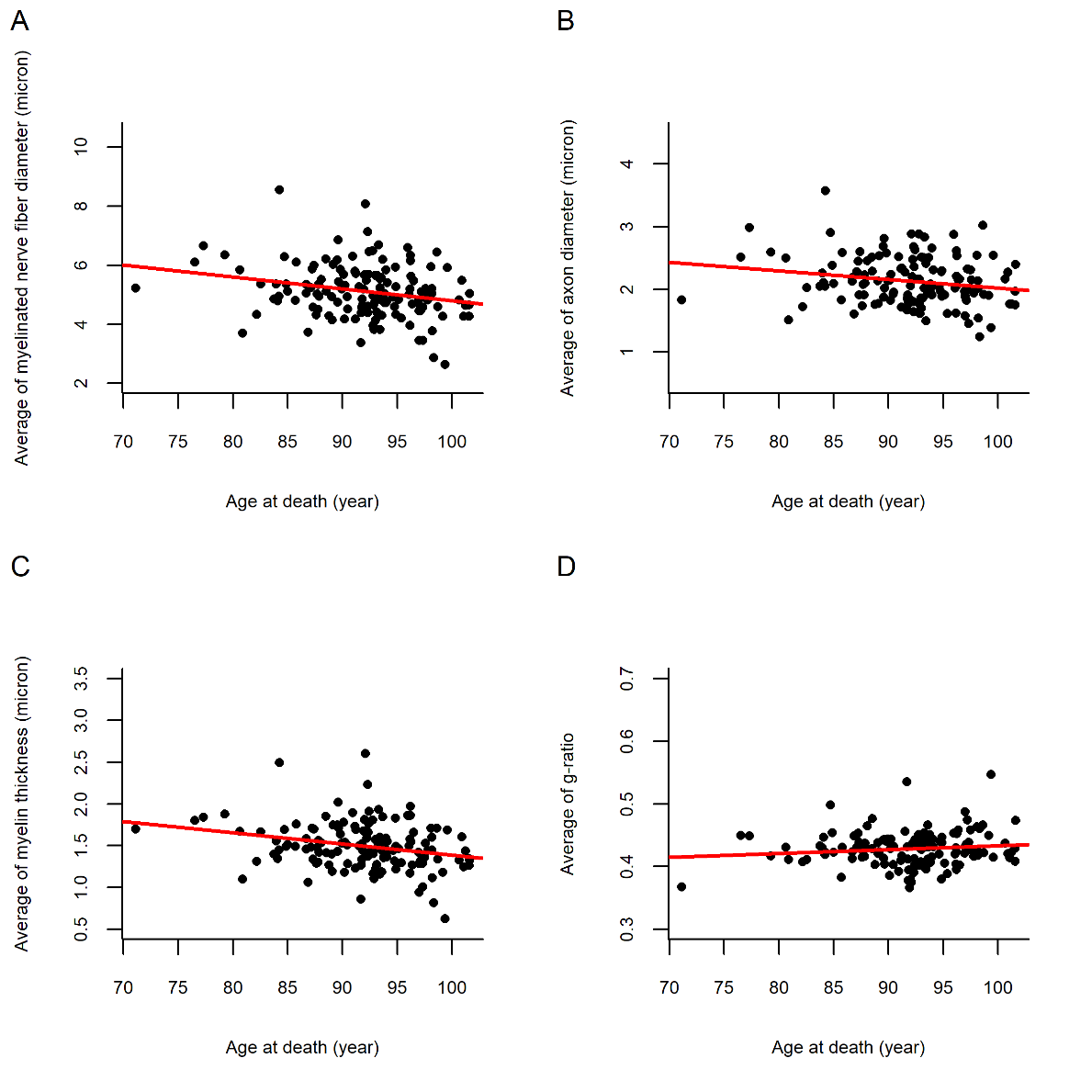
**

The 4 scatter plots illustrate the association between age at death and person-specific averages of myelinated nerve fibers diameter (A), axonal diameter (B), myelin thickness (C), and g-ratio (D). The person-specific averages were calculated from 721540 myelinated nerve fibers, which were prepared by excluding 32707 out of the pool of 754247 myelinated nerve fibers that had a diameter < 1 µm. Each dot represents a participant’s age at death (x-axis) and one of the person-specific averages of the morphometric characteristics. The red lines are derived from linear regression models including 140 participants.

**Supplementary Table 11. Association of age at death with the percentage of myelinated nerve fibers of different diameters, calculated after removing 32707 myelinated nerve fibers with a diameter < 1 µm.**

| **Model terms** | **Estimate (SE), p-value** |
| --- | --- |
| Intercept | 1.875 (0.041), <0.001 |
| Age at death | 0.000 (0.006), 0.953 |
| Sex | 0.025 (0.055), 0.647 |
| Percent of myelinated nerve fibers 2 to <4µm / Percent of myelinated nerve fibers <2µm | REF. |
| Percent of myelinated nerve fibers 4 to <6µm / Percent of myelinated nerve fibers <2µm | -0.660 (0.070), <0.001 |
| Percent of myelinated nerve fibers 6 to <8µm / Percent of myelinated nerve fibers <2µm | -1.605 (0.070), <0.001 |
| Percent of myelinated nerve fibers 8 to <10µm / Percent of myelinated nerve fibers <2µm | -1.758 (0.080), <0.001 |
| Percent of myelinated nerve fibers ≥10µm / Percent of myelinated nerve fibers <2µm | -1.621 (0.094), <0.001 |
| Age × (Percent of myelinated nerve fibers 4 to <6µm / Percent of myelinated nerve fibers <2µm) | -0.007 (0.012), 0.551 |
| Age × (Percent of myelinated nerve fibers 6 to <8µm / Percent of myelinated nerve fibers <2µm) | -0.018 (0.012), 0.136 |
| Age × (Percent of myelinated nerve fibers 8 to <10µm / Percent of myelinated nerve fibers <2µm) | -0.035 (0.014), 0.011 |
| Age × (Percent of myelinated nerve fibers ≥10µm / Percent of myelinated nerve fibers <2µm) | -0.039 (0.016), 0.015 |

**Supplementary Figure 10. Association of age at death with density of myelinated nerve fibers, calculated after removing 32707 myelinated nerve fibers with a diameter < 1 µm.**

**
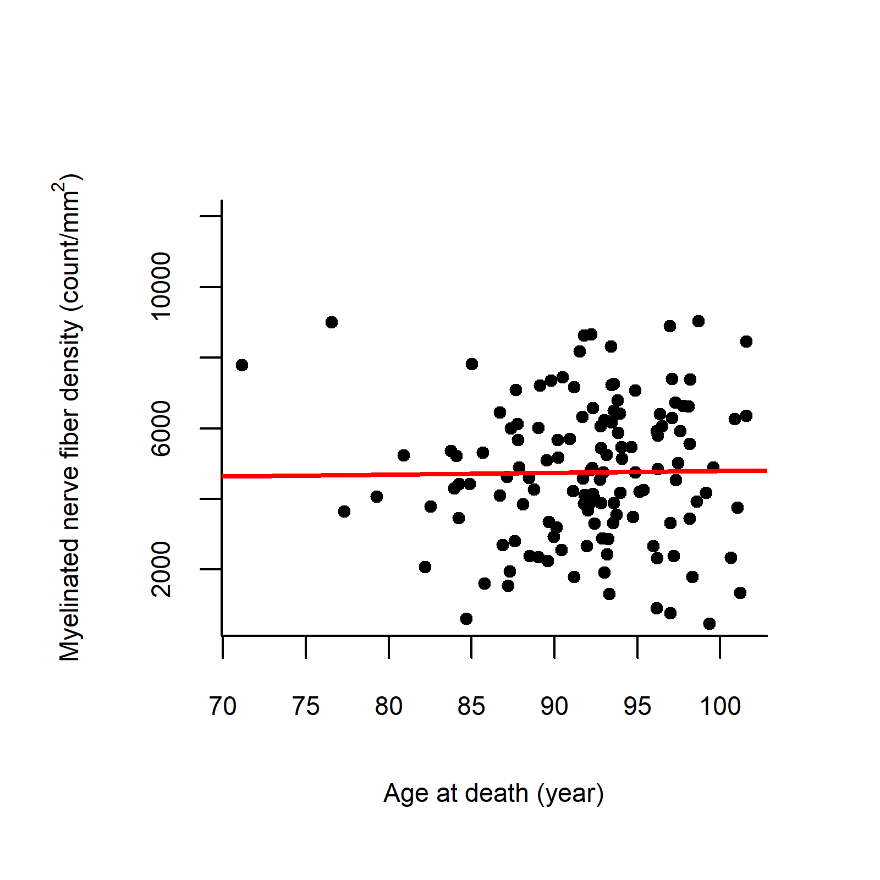
**

In a linear regression model, myelinated nerve fiber density was the outcome and age at death as the model term, controlled for sex. Myelinated nerve fiber density was calculated after excluding myelinated nerve fibers with a diameter < 1 µm. The sample size was 134 because the nerve area was missed in 9 participants. Each dot represents the myelinated nerve fiber density of the tibial nerve and age at death of a participant.

**Supplementary Table 12. Association of age at death with g-ratio of the myelinated nerve fibers classified by their diameter after removing 32707 myelinated nerve fibers with a diameter < 1 µm.**

| **Model terms** | **Estimate (SE), p-value** |
| --- | --- |
| Intercept | 0.422 (0.003), <0.001 |
| Age at death | 0.0013 (0.0004), 0.002 |
| Sex | -0.006 (0.002), 0.005 |
| Myelinated nerve fibers <2µm | 0.215 (0.008), <0.001 |
| Myelinated nerve fibers 2 to <4 µm | -0.009 (0.003), 0.004 |
| Myelinated nerve fibers 4 to <6 µm | -0.002 (0.003), 0.475 |
| Myelinated nerve fibers 6 to <8µm | -0.011 (0.004), 0.012 |
| Myelinated nerve fibers 8 to <10µm | -0.023 (0.004), <0.001 |
| Myelinated nerve fibers ≥10µm | **REF** |
| Age×Myelinated nerve fibers <2µm | -0.004 (0.001), 0.003 |
| Age×Myelinated nerve fibers 2 to <4µm | -0.0016 (0.0005), 0.002 |
| Age×Myelinated nerve fibers 4 to <6µm | -0.0011 (0.0005), 0.038 |
| Age×Myelinated nerve fibers 6 to <8µm | 0.0006 (0.0008), 0.443 |
| Age×Myelinated nerve fibers 8 to <10µm | 0.0015 (0.0006), 0.020 |

**Supplementary Table 13. Descriptive statistics of the myelinated nerve fibers after removing 66550 myelinated nerve fibers with an axon diameter < 0.8 µm.**

| **Characteristics** | **Mean (SD) or count** |
| --- | --- |
| **The myelinated nerve fibers altogether** |  |
| Number of the examined myelinated nerve fibers | 687697 |
| Diameter of myelinated nerve fiber, (μm) | 5.2 (3.0) |
| Axon diameter, (μm) | 2.2 (1.4) |
| Myelin thickness, (μm) | 1.5 (1.0) |
| G-ratio | 0.44 (0.13) |
| **Characteristics at person-level** |  |
| Number of the myelinated nerve fibers per participant | 4912.1 (3153.7) |
| Person-specific average of myelinated nerve fiber diameter, (μm) | 5.2 (0.9) |
| Person-specific average of axon diameter, (μm) | 2.2 (0.4) |
| Person-specific average of myelin thickness, (μm) | 1.5 (0.3) |
| Person-specific average of g-ratio | 0.45 (0.03) |
| Myelinated nerve fiber density, (count/mm^2^) | 4544.4 (1883.2) |

**Supplementary Figure 11. Distribution of morphometric characteristics of myelinated nerve fibers (n=687697) after removing 66550 myelinated nerve fibers with an axon diameter < 0.8 µm.**


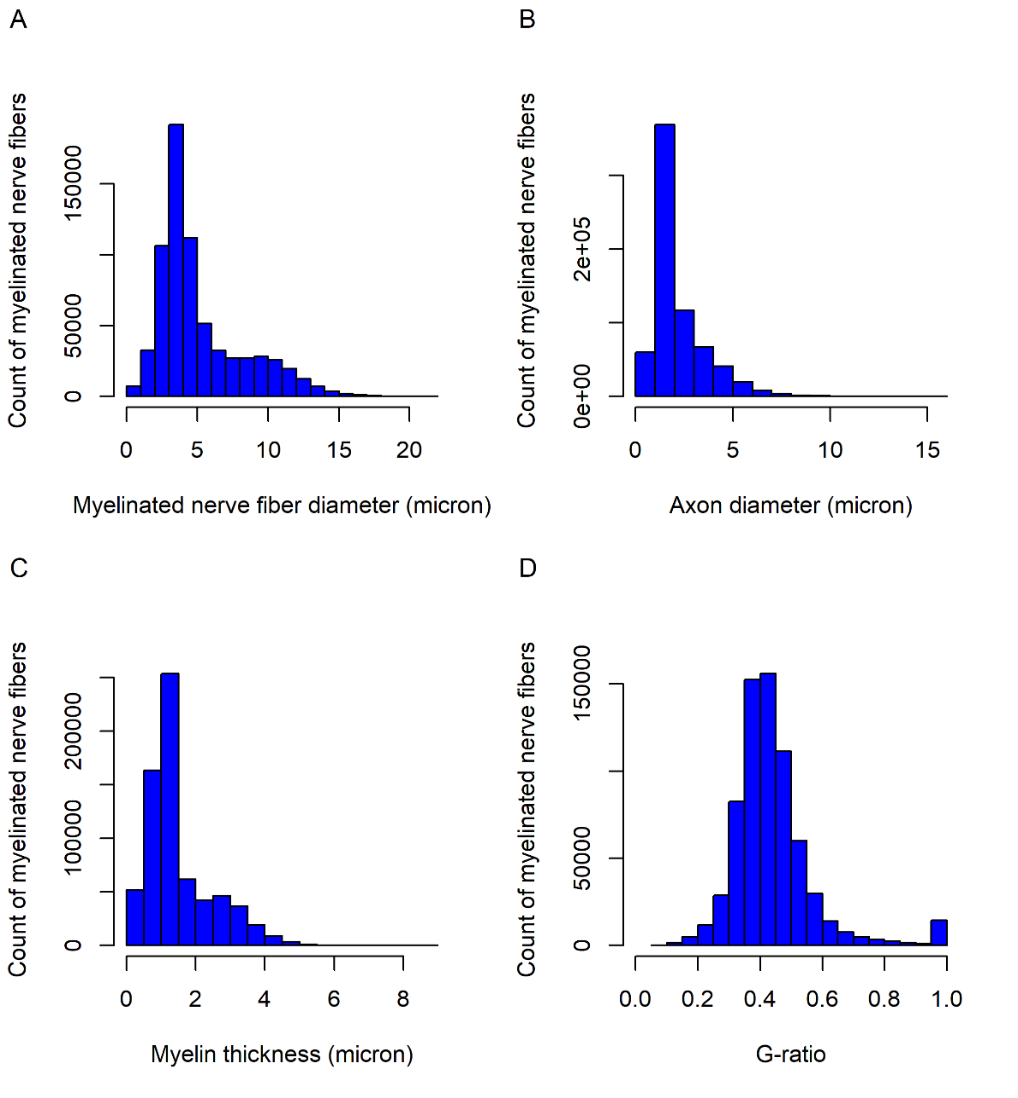


Histogram of 687697 myelinated nerve fibers illustrating their distribution by the diameter of the myelinated nerve fiber (A), axonal diameter (B), myelin thickness (C), and g-ratio (D). The 687697 myelinated nerve fibers from selected from the pool of 754247 nerve fibers after excluding myelinated nerve fibers with an axonal diameter < 0.8 µm.

**Supplementary Figure 12. Associations of axon diameter with myelin thickness after removing 66550 myelinated nerve fibers with an axon diameter < 0.8 µm.**


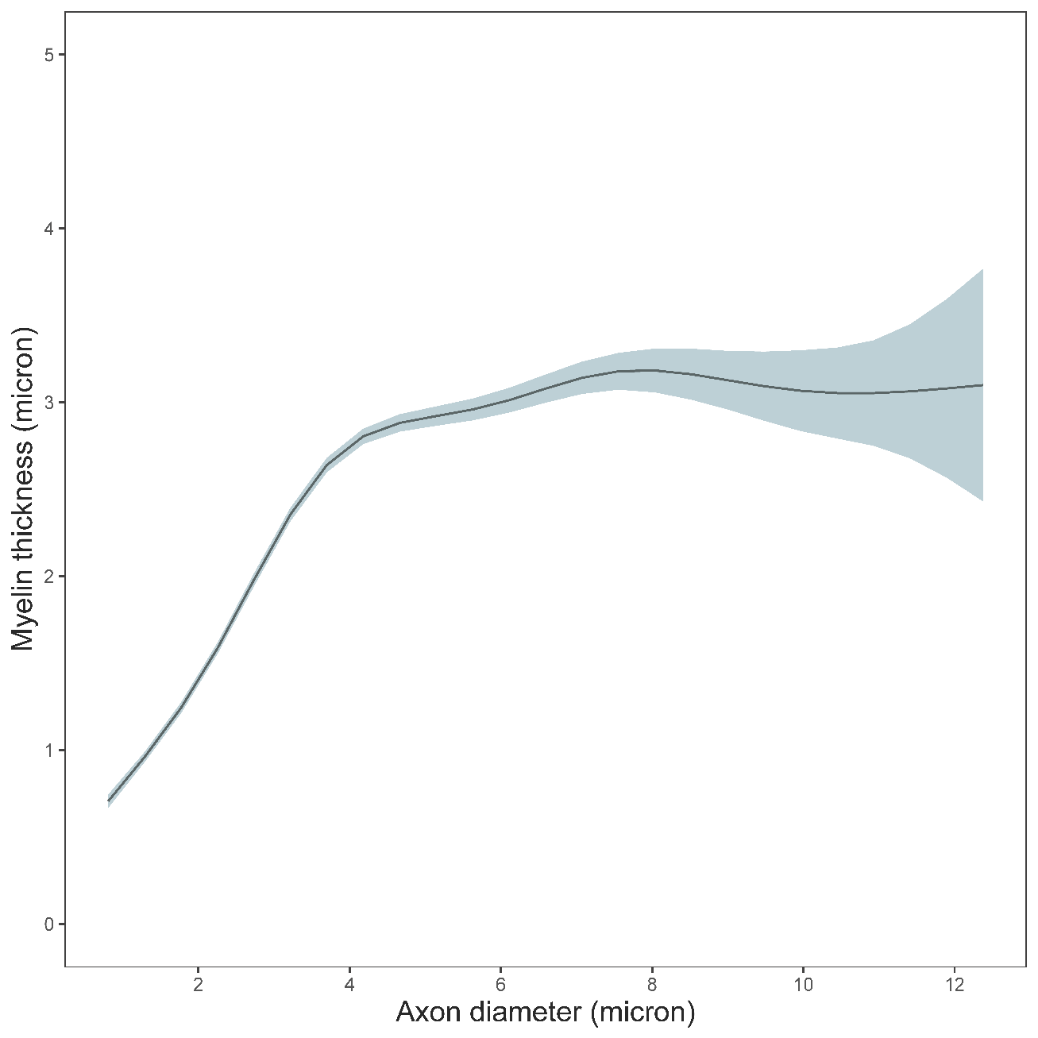


Association between axon diameter and myelin thickness derived from a generalized additive mixed model, using the *gamm* function from *mgcv* package^1^ in R, analyzing 14000 myelinated nerve fibers, which included 100 randomly selected myelinated nerve fibers from each of 140 participants. The 14000 myelinated nerve fibers were selected from 687697 myelinated nerve fibers, which were prepared by excluding 66550 out of the pool of 754247 myelinated nerve fibers that had an axonal diameter < 0.8 µm. The Y-axis is the estimated myelin thickness. The shaded blue area around the curve indicates 95% confidence interval of myelin thickness.

**Supplementary Table 14. Associations of age at death with person-specific averages of the morphometric characteristics of the tibial nerve, calculated after removing 66550 myelinated nerve fibers with an axon diameter < 0.8 µm.**

| **Morphometric characteristics** | **Associations of age at death** | |
| --- | --- | --- |
|  | **Spearman correlation coefficient, p-value** | **Estimate (SE), p-value** |
| **Person-specific average of myelinated nerve fiber diameter** | -0.24, 0.005 | -0.042 (0.014), 0.003 |
| **Person-specific average of axon diameter** | -0.17, 0.043 | -0.015 (0.006), 0.016 |
| **Person-specific average of myelin thickness** | -0.26, 0.002 | -0.014 (0.004), 0.002 |
| **Person-specific average of g-ratio** | 0.09, 0.31 | 0.0006 (0.0005), 0.20 |
| **Myelinated nerve fiber density** | 0.08, 0.36 | 6.77 (31.04), 0.83 |

In 5 separate linear regression models, each of the person-specific averages of the morphometric characteristics of the tibial nerve was the outcome, and age at death and sex were the model terms. The person-specific averages of the morphometric characteristics were calculated after exclusion of 66550 myelinated nerve fibers with a diameter < 0.8 µm. Age at death was associated with smaller myelinated nerve fibers, smaller axons, and thinner myelin, but g-ratio and myelinated nerve fiber density were not related to age at death.

**Supplementary Figure 13. Scatter plots of age at death in relation to person-specific averages of myelinated nerve fiber characteristics, calculated after removing 66550 myelinated nerve fibers with an axon diameter < 0.8 µm.**

**
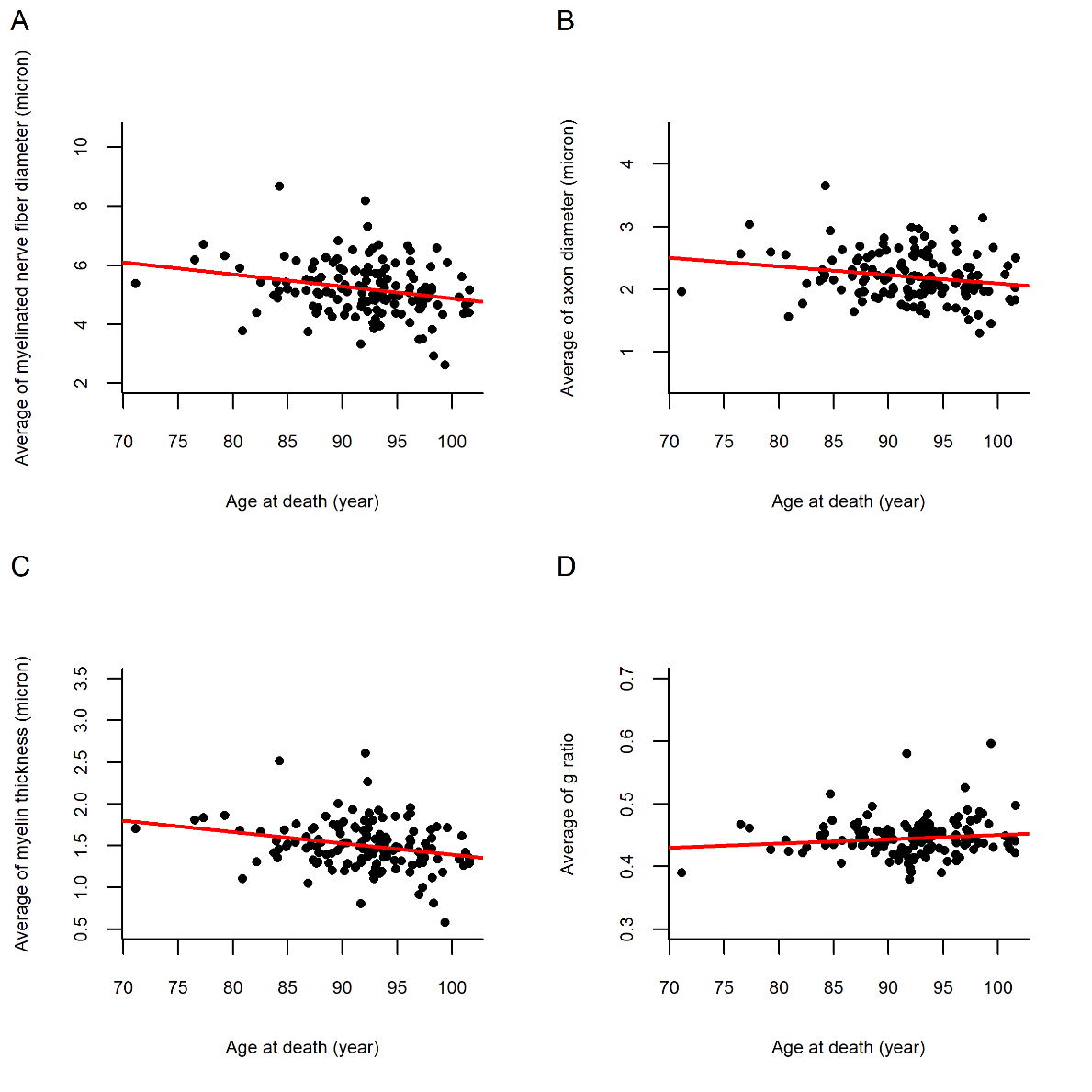
**

The 4 scatter plots illustrate the association between age at death and person-specific averages of myelinated nerve fibers diameter (A), axonal diameter (B), myelin thickness (C), and g-ratio (D). The person-specific averages were calculated from 687697 myelinated nerve fibers, which were prepared by excluding 66550 out of the pool of 754247 myelinated nerve fibers that had an axonal diameter < 0.8 µm. Each dot represents a participant’s age at death (x-axis) and one of the person-specific averages of the morphometric characteristics. The red lines are derived from linear regression models including 140 participants.

**Supplementary Table 15. Association of age at death with the percentage of myelinated nerve fibers of different diameters, calculated after removing 66550 myelinated nerve fibers with an axon diameter < 0.8 µm.**

| **Model terms** | **Estimate (SE), p-value** |
| --- | --- |
| Intercept | 1.885 (0.046), <0.001 |
| Age at death | 0.002 (0.007), 0.759 |
| Sex | 0.027 (0.057), 0.629 |
| Percent of myelinated nerve fibers 2 to <4µm / Percent of myelinated nerve fibers <2µm | REF. |
| Percent of myelinated nerve fibers 4 to <6µm / Percent of myelinated nerve fibers <2µm | -0.609 (0.074), <0.001 |
| Percent of myelinated nerve fibers 6 to <8µm / Percent of myelinated nerve fibers <2µm | -1.543 (0.072), <0.001 |
| Percent of myelinated nerve fibers 8 to <10µm / Percent of myelinated nerve fibers <2µm | -1.685 (0.080), <0.001 |
| Percent of myelinated nerve fibers ≥10µm / Percent of myelinated nerve fibers <2µm | -1.545 (0.095), <0.001 |
| Age × (Percent of myelinated nerve fibers 4 to <6µm / Percent of myelinated nerve fibers <2µm) | -0.006 (0.012), 0.600 |
| Age × (Percent of myelinated nerve fibers 6 to <8µm / Percent of myelinated nerve fibers <2µm) | -0.018 (0.012), 0.138 |
| Age × (Percent of myelinated nerve fibers 8 to <10µm / Percent of myelinated nerve fibers <2µm) | -0.035 (0.013), 0.009 |
| Age × (Percent of myelinated nerve fibers ≥10µm / Percent of myelinated nerve fibers <2µm) | -0.038 (0.016), 0.016 |

**Supplementary Figure 14. Association of age at death with density of myelinated nerve fibers, calculated after removing 66550 myelinated nerve fibers with an axon diameter < 0.8 µm.**

**
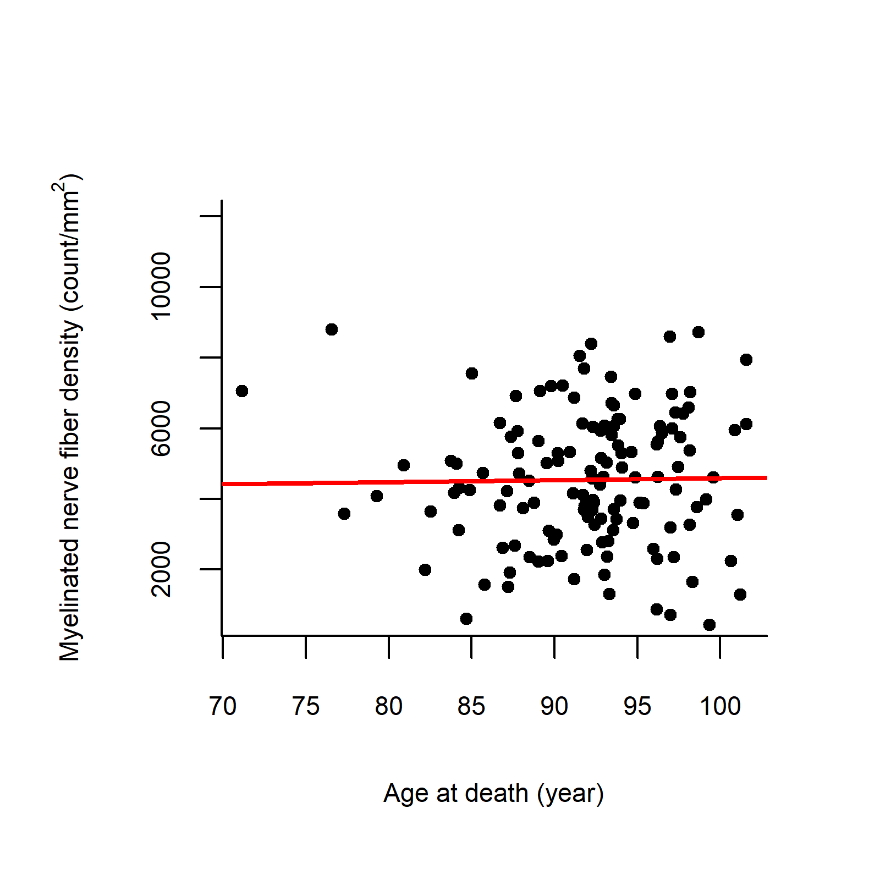
**

In a linear regression model, myelinated nerve fiber density was the outcome and age at death as the model term, controlled for sex. Myelinated nerve fiber density was calculated after excluding myelinated nerve fibers with an axonal diameter < 0.8 µm. The sample size was 134 because the nerve area was missed in 9 participants. Each dot represents the myelinated nerve fiber density of the tibial nerve and age at death of a participant.

**Supplementary Table 16. Association of age at death with g-ratio of the myelinated nerve fibers classified by their diameter after removing 66550 myelinated nerve fibers with an axon diameter < 0.8 µm.**

| **Model terms** | **Estimate (SE), p-value** |
| --- | --- |
| Intercept | 0.425 (0.003), <0.001 |
| Age at death | 0.0013 (0.0005), 0.007 |
| Sex | -0.007 (0.002), <0.001 |
| Myelinated nerve fibers <2µm | 0.347 (0.007), <0.001 |
| Myelinated nerve fibers 2 to <4 µm | 0.003 (0.003), 0.253 |
| Myelinated nerve fibers 4 to <6 µm | 0.001 (0.003), 0.654 |
| Myelinated nerve fibers 6 to <8µm | -0.012 (0.004), 0.005 |
| Myelinated nerve fibers 8 to <10µm | -0.014 (0.004), <0.001 |
| Myelinated nerve fibers ≥10µm | **REF** |
| Age×Myelinated nerve fibers <2µm | -0.003 (0.001), 0.010 |
| Age×Myelinated nerve fibers 2 to <4µm | -0.0021 (0.0005), <0.001 |
| Age×Myelinated nerve fibers 4 to <6µm | -0.0012 (0.0006), 0.027 |
| Age×Myelinated nerve fibers 6 to <8µm | -0.0002 (0.0007), 0.807 |
| Age×Myelinated nerve fibers 8 to <10µm | -0.0005 (0.0006), 0.439 |

**Supplementary references**

1. Wood S. Package ‘mgcv.’ Published online 2023. Accessed July 11, 2024. https://cran.r-project.org/web/packages/mgcv/mgcv.pdf
